# Trends and disparities in depression-related mortality among older adults in the United States, 1999–2020

**DOI:** 10.3389/fpubh.2026.1908154

**Published:** 2026-10-28

**Authors:** Tong Feng, Pengcheng Zheng, Qingyuan Li, Ding Han, Ran Duan

**Affiliations:** 1 Department of Respiratory and Critical Care Medicine, Deyang People’s Hospital, Deyang, China; 2 School of Clinical Medicine, Chengdu Medical College, Chengdu, China; 3 Department of Respiratory and Critical Care Medicine, The First Affiliated Hospital of Chengdu Medical College, Chengdu, China; 4 Laboratory of Geriatric Respiratory Diseases of Sichuan Higher Education Institute, Chengdu, China; 5 Department of Oncology, The First Affiliated Hospital of Chengdu Medical College, Chengdu, China

**Keywords:** CDC WONDER, depression, disparities, mortality, older adults

## Abstract

**Background:**

National trends in mortality with depression recorded as the underlying cause among older adults remain insufficiently characterized. We examined recorded mortality and demographic and geographic disparities among U.S. adults aged ≥65 years during 1999–2020.

**Methods:**

CDC WONDER Underlying Cause of Death data were used to identify deaths with ICD-10 F32 or F33 as the underlying cause. Age-adjusted mortality rates (AAMRs) per 100,000 population and age-specific rates were calculated. Joinpoint regression assessed temporal trends, ARIMA generated exploratory projections through 2030, and Frontier analysis provided descriptive state-level benchmarking. Sensitivity analyses examined any mention of F32/F33 in multiple-cause-of-death records and trends after excluding 2020.

**Results:**

The primary analysis identified 11,858 deaths, with an overall AAMR of 1.29 per 100,000. Higher recorded rates were observed among women, non-Hispanic and White older adults, nonmetropolitan residents, and residents of the Midwest. Overall AAMR declined (AAPC, −3.26%; *p* < 0.001), most sharply during 2003–2007; the 2018–2020 increase was not statistically significant. A long-term decline remained after excluding 2020. The broader multiple-cause definition identified 236,831 deaths, 19.97 times the primary count, and showed a different late-period pattern. ARIMA projected declining rates. Frontier analysis showed substantial state-level heterogeneity.

**Conclusion:**

Mortality with depression recorded as the underlying cause declined, but the broader any-mention definition yielded different trends. Continued surveillance, equitable recognition and documentation, and culturally responsive geriatric mental health care remain important.

## Graphical abstract

**Figure:**
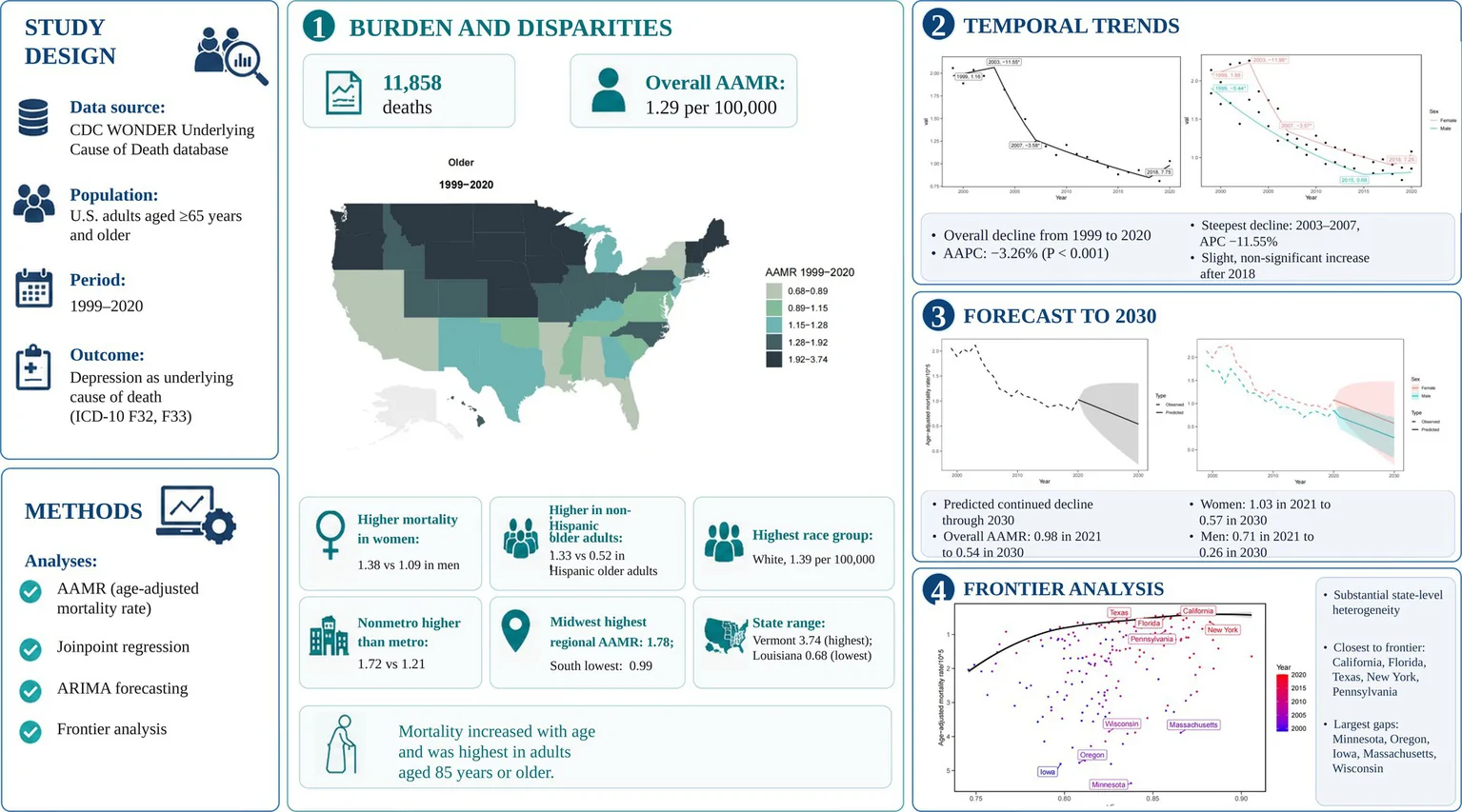

## Introduction

Late-life depression, commonly defined as depression occurring among adults aged 65 years and older, represents an increasingly important public health concern in the context of population aging. The global population is aging rapidly, and older adults are particularly vulnerable to mental health conditions because of chronic illness, functional decline, bereavement, social isolation, loneliness, reduced income, retirement-related loss of purpose, and limited access to supportive services (1, 2). Although depression is not an inevitable part of aging, it is frequently underrecognized and undertreated in older adults, partly because depressive symptoms may overlap with somatic complaints, cognitive impairment, sleep disturbance, frailty, disability, and multimorbidity.

Beyond its direct psychiatric burden, depression in later life is associated with substantial clinical and public health consequences (3, 4). Biological pathways, including hypothalamic–pituitary–adrenal axis dysregulation, chronic inflammation, autonomic dysfunction, vascular injury, and sleep disruption, may contribute to adverse health outcomes among older adults with depression (5, 6). Behavioral and social mechanisms, including reduced physical activity, poor nutrition, medication nonadherence, delayed care seeking, social withdrawal, and diminished self-care, may further increase morbidity and mortality risk (7, 8). These mechanisms are especially relevant in adults aged 65 years and older, who commonly live with coexisting cardiovascular, metabolic, neurologic, and functional conditions (9).

Despite growing attention to geriatric mental health, national mortality patterns associated with documented depression among older adults remain insufficiently characterized. Prior population-based studies using the Centers for Disease Control and Prevention Wide-ranging Online Data for Epidemiologic Research database have evaluated depression-associated mortality in relation to cardiovascular disease and other chronic conditions, but relatively limited attention has been given to long-term depression-related mortality specifically among adults aged 65 years and older (10, 11). In particular, trends and disparities by sex, race and ethnicity, age subgroup, geographic region, state, and urban–rural status remain poorly defined (12). These data provide a valuable national surveillance signal for evaluating temporal trends and identifying populations with higher recorded mortality.

Therefore, this study used CDC WONDER data to examine mortality with depression recorded as the underlying cause among U.S. adults aged 65 years and older from 1999 to 2020. Annual age-adjusted mortality rates per 100,000 population were calculated and stratified by sex, race and ethnicity, U.S. Census region, state, and urban–rural status; age-specific rates were examined separately. Joinpoint regression identified changes in temporal trends, and autoregressive integrated moving average (ARIMA) models provided exploratory mortality projections through 2030. Frontier analysis described state-level variation relative to an empirical lower-mortality benchmark, without attributing gaps to health policy or preventable mortality. Sensitivity analyses using multiple-cause-of-death records and excluding 2020 assessed the dependence of conclusions on case definition and the final observation year. Together, these complementary analyses support descriptive geriatric mental health surveillance and the identification of priorities for further investigation.

## Methods

### Study design and data source

We conducted a descriptive, population-based retrospective surveillance study using the Centers for Disease Control and Prevention Wide-ranging Online Data for Epidemiologic Research (CDC WONDER) Underlying Cause of Death database (13). The database contains mortality records derived from death certificates for U.S. residents and corresponding population estimates. The study period extended from January 1, 1999, to December 31, 2020.

Because the study used publicly available, deidentified, aggregate data, institutional review board approval and informed consent were not required.

The study was descriptive rather than etiological. Aggregated mortality records were not linked to individual comorbidities, antidepressant treatment, or adherence, and no multivariable adjustment was performed for mental health workforce supply, prescribing patterns, Medicaid policies, or other area-level covariates.

### Study population and ascertainment of recorded depression-related deaths

The study population comprised U.S. residents aged 65 years and older whose deaths were recorded in CDC WONDER between January 1, 1999, and December 31, 2020. Eligibility for the primary analysis required death during this period, age ≥65 years at death, U.S. residency, and depression recorded as the underlying cause of death. The underlying cause was the disease or condition initiating the sequence of events leading directly to death.

Depression was identified using the International Classification of Diseases, Tenth Revision (ICD-10) codes F32 (depressive episode) and F33 (recurrent depressive disorder) (14).

Deaths outside the study period, deaths among people younger than 65 years, and deaths with an underlying cause other than F32 or F33 were excluded from the primary analysis. Deaths mentioning depression only as a contributing or associated condition were excluded from the primary analysis but could be included in the separate any-mention sensitivity analysis. Throughout the manuscript, primary depression-related mortality denotes recorded underlying-cause mortality, not all deaths among people with depression or all deaths causally attributable to depression.

### Demographic and geographic variables

Mortality data were extracted by calendar year and stratified according to sex, age group, race, Hispanic origin, U.S. Census region, state of residence, and urbanization status.

Age-specific analyses were conducted for adults aged 65–74, 75–84, and 85 years or older. Sex was categorized as female or male. Race was categorized as White, Black or African American, American Indian or Alaska Native, and Asian or Pacific Islander. Hispanic origin was analyzed separately as Hispanic or Latino and non-Hispanic or non-Latino. U.S. Census regions were categorized as Northeast, Midwest, South, and West. Urbanization status was classified as metropolitan or nonmetropolitan according to the categories available in CDC WONDER.

The 65–74-, 75–84-, and ≥85-year categories provided a consistent and interpretable framework for comparisons across stages of later life and across calendar years and sexes. Ten-year intervals for ages 65–84 balanced detail against statistical stability; the open-ended ≥85-year group avoided excessive fragmentation of the oldest population.

### Mortality-rate estimation

The numbers of deaths, population denominators, crude mortality rates, age-specific mortality rates, and age-adjusted mortality rates (AAMRs) were obtained from CDC WONDER. Mortality rates were expressed as deaths per 100,000 population. AAMRs were calculated using direct standardization to the 2000 U.S. standard population and are presented with corresponding 95% confidence intervals (CIs).

Age-specific mortality rates were reported for the three prespecified age groups. AAMRs were used for comparisons across calendar years and demographic or geographic subgroups to account for differences in population age structure.

In accordance with the reporting rules used for the CDC WONDER extracts, aggregate cells containing 1–9 deaths were suppressed for confidentiality, while rates based on fewer than 20 deaths were considered statistically unreliable and were not interpreted as stable estimates. These restrictions applied to the reporting and interpretation of aggregate estimates, not to the eligibility of individual deaths. Missing or unreliable subgroup rates were not treated as zero.

### Temporal trend analysis

Temporal trends in annual AAMRs were evaluated using the Joinpoint Regression Program, version 5.4.0, released in April 2025 by the Surveillance Research Program of the National Cancer Institute (15, 16).

Piecewise log-linear regression models were fitted to annual AAMRs to identify inflection points at which the direction or magnitude of the mortality trend changed. Annual percent changes (APCs) and corresponding 95% CIs were calculated for each trend segment. Average annual percent changes (AAPCs) and corresponding 95% CIs were calculated to summarize the overall trends during the study period.

Using the default settings of Joinpoint version 5.4.0, the optimal number of joinpoints was selected using the weighted Bayesian information criterion (WBIC), and confidence intervals were calculated using the empirical quantile method. Statistical significance was assessed using two-sided tests, with a *p* value of less than 0.05 considered statistically significant.

Joinpoint analyses were conducted for the overall population and stratified by sex, urbanization status, and U.S. Census region.

### Sensitivity analysis excluding 2020

To evaluate the influence of the first COVID-19 pandemic year, separate unsegmented log-linear models of annual AAMRs were fitted for 1999–2020 and 1999–2019, overall and by sex. APCs and 95% confidence intervals were obtained by exponentiating the calendar-year coefficients, using Newey–West heteroskedasticity- and autocorrelation-consistent standard errors with two annual lags (17).

### Sensitivity analysis using multiple-cause-of-death data

To assess the impact of the primary case definition, we analyzed CDC WONDER Multiple Cause of Death data for 1999–2020. Deaths among U.S. adults aged ≥65 years were selected when either ICD-10 F32 or F33 was mentioned among the recorded causes, without restricting the underlying cause. Annual death counts and population denominators were combined across the 65–74-, 75–84-, and ≥85-year groups to calculate crude mortality rates.

Directly standardized multiple-cause rates were additionally calculated using 2000 U.S. standard population weights for the three age groups, renormalized within ages ≥65 years. Approximate 95% CIs were derived from Poisson variances using a normal approximation.

### ARIMA-based forecasting

Autoregressive integrated moving average (ARIMA) models were used to generate exploratory projections of annual AAMRs (18, 19). The overall, female, and male series each comprised 22 annual observations from 1999 through 2020. The annual series were treated as nonseasonal.

The primary forecasts were generated in R using the auto.arima() function in the forecast package (20). A separate independent Python refit was used to characterize selected nonseasonal model specifications with drift, corrected Akaike information criterion (AICc) values, and residual diagnostics.

Forecasts for 2021–2030 are reported with model-based 95% prediction intervals (PIs). For the independent refit, residual autocorrelation and partial autocorrelation functions (ACF and PACF) were inspected, and a Ljung–Box test at lag 5 was applied to the 21 post-differencing innovations, with degrees of freedom adjusted for fitted autoregressive and moving-average terms (p + q). One-year-ahead rolling-origin forecasts for 2015–2020 were compared with damped-trend exponential smoothing (ETS) and a naive last-observation benchmark using mean absolute error (MAE) and root mean squared error (RMSE).

### Sociodemographic index and frontier analysis

State-level sociodemographic index (SDI) values were linked to mortality data by state and calendar year. SDI is a composite indicator expressed on a scale from 0 to 1, with higher values representing higher levels of sociodemographic development. The index incorporates income per capita, educational attainment, and fertility among individuals younger than 25 years.

The relationship between state-level SDI and recorded underlying-cause AAMR was evaluated using nonlinear smoothing. Frontier analysis was performed in R to describe an empirical lower-mortality benchmark at a given SDI level.

The frontier gap was defined as the difference between observed AAMR and the estimated lower-mortality frontier value. Smaller gaps indicated closer proximity to the empirical benchmark; larger gaps indicated greater observed deviation, not necessarily preventable mortality. States were ranked by their distance from the estimated frontier, and the five states closest to the frontier and the five with the largest gaps were highlighted graphically.

To contextualize these comparisons, SDI in 1999 and 2020, the arithmetic mean of the 22 annual SDI values, and the absolute endpoint change were summarized for the 10 highlighted states.

### Statistical analysis

The primary Joinpoint analyses used the Joinpoint Regression Program, version 5.4.0. Primary ARIMA forecasting, SDI smoothing, and Frontier analysis used R version 4.5.1 (R Foundation for Statistical Computing, Vienna, Austria). Two-sided *p* values <0.05 were considered statistically significant. Rate estimates and confidence or prediction intervals are displayed to two decimal places, with additional precision retained for model diagnostics, forecast-error metrics, and SDI.

## Results

### Overall mortality burden and demographic disparities

From 1999 to 2020, a total of 11,858 deaths with depression recorded as the underlying cause were identified among adults aged 65 years and older in the United States, corresponding to an overall age-adjusted mortality rate (AAMR) of 1.29 per 100,000 population (95% CI, 1.27–1.32). Substantial geographic and demographic disparities in recorded mortality were observed (Table 1).

**Table 1:** Recorded mortality with depression as the underlying cause among U.S. adults aged ≥65 years, by demographic and geographic characteristics, 1999–2020.

Characteristic	Deaths/person-years	AAMR (95% CI)
Overall	11,858/928,476,665	1.29 (1.27–1.32)
Census region		
Midwest	3,819/207,118,111	1.78 (1.73–1.84)
Northeast	2,491/179,714,300	1.32 (1.26–1.37)
South	3,243/343,442,253	0.99 (0.96–1.02)
West	2,305/198,202,001	1.20 (1.15–1.25)
Sex		
Female	8,079/527,632,126	1.38 (1.35–1.41)
Male	3,779/400,844,539	1.09 (1.05–1.12)
Hispanic origin		
Hispanic or Latino	285/65,124,722	0.52 (0.46–0.58)
Not Hispanic or Latino	11,552/863,351,943	1.33 (1.30–1.35)
Race		
American Indian or Alaska Native	41/6,051,868	0.88 (0.62–1.19)
Asian or Pacific Islander	127/35,919,615	0.44 (0.36–0.51)
Black or African American	394/83,274,492	0.54 (0.48–0.59)
White	11,296/803,230,690	1.39 (1.36–1.42)
Urbanization		
Metropolitan	9,005/760,496,648	1.21 (1.18–1.23)
Nonmetropolitan	2,853/167,978,547	1.72 (1.66–1.78)

Deaths are counts with depression (ICD-10 F32/F33) recorded as the underlying cause. Denominators are sums of annual population estimates over 1999–2020 (person-years), not counts of unique individuals. AAMRs and 95% confidence intervals are per 100,000 population and use the 2000 U.S. standard population. Race and Hispanic origin were analyzed separately. Hispanic-origin rows do not sum to all deaths in the supplied table (285 + 11,552 = 11,837 vs 11,858); no missing category has been imputed. Urbanization denominators are retained as supplied. AAMR, age-adjusted mortality rate; CI, confidence interval.

The Midwest had the highest recorded regional AAMR at 1.78 per 100,000 population (95% CI, 1.73–1.84), followed by the Northeast (1.32; 95% CI, 1.26–1.37), the West (1.20; 95% CI, 1.15–1.25), and the South (0.99; 95% CI, 0.96–1.02). Women accounted for 8,079 deaths (68.1%) and had a higher recorded AAMR than men (1.38 [95% CI, 1.35–1.41] vs. 1.09 [95% CI, 1.05–1.12] per 100,000 population). The AAMR was also higher in nonmetropolitan areas than in metropolitan areas (1.72 [95% CI, 1.66–1.78] vs. 1.21 [95% CI, 1.18–1.23]).

Differences in recorded underlying-cause mortality were observed by race and ethnicity. Non-Hispanic older adults had a higher recorded AAMR than Hispanic or Latino older adults (1.33 [95% CI, 1.30–1.35] vs. 0.52 [95% CI, 0.46–0.58]). White older adults had the highest recorded AAMR (1.39; 95% CI, 1.36–1.42), followed by American Indian or Alaska Native older adults (0.88; 95% CI, 0.62–1.19), Black or African American older adults (0.54; 95% CI, 0.48–0.59), and Asian or Pacific Islander older adults (0.44; 95% CI, 0.36–0.51).

State-level recorded AAMRs varied by more than fivefold among states with reportable estimates (Figure 1 and Supplementary Table S1). The highest AAMRs were observed in Vermont (3.74 per 100,000 population), New Hampshire (3.14), Wyoming (3.07), North Dakota (2.98), and Oregon (2.91). The lowest AAMRs were observed in Louisiana (0.68), Florida (0.74), California (0.76), Alabama (0.77), and New York (0.82).

**Figure 1:**
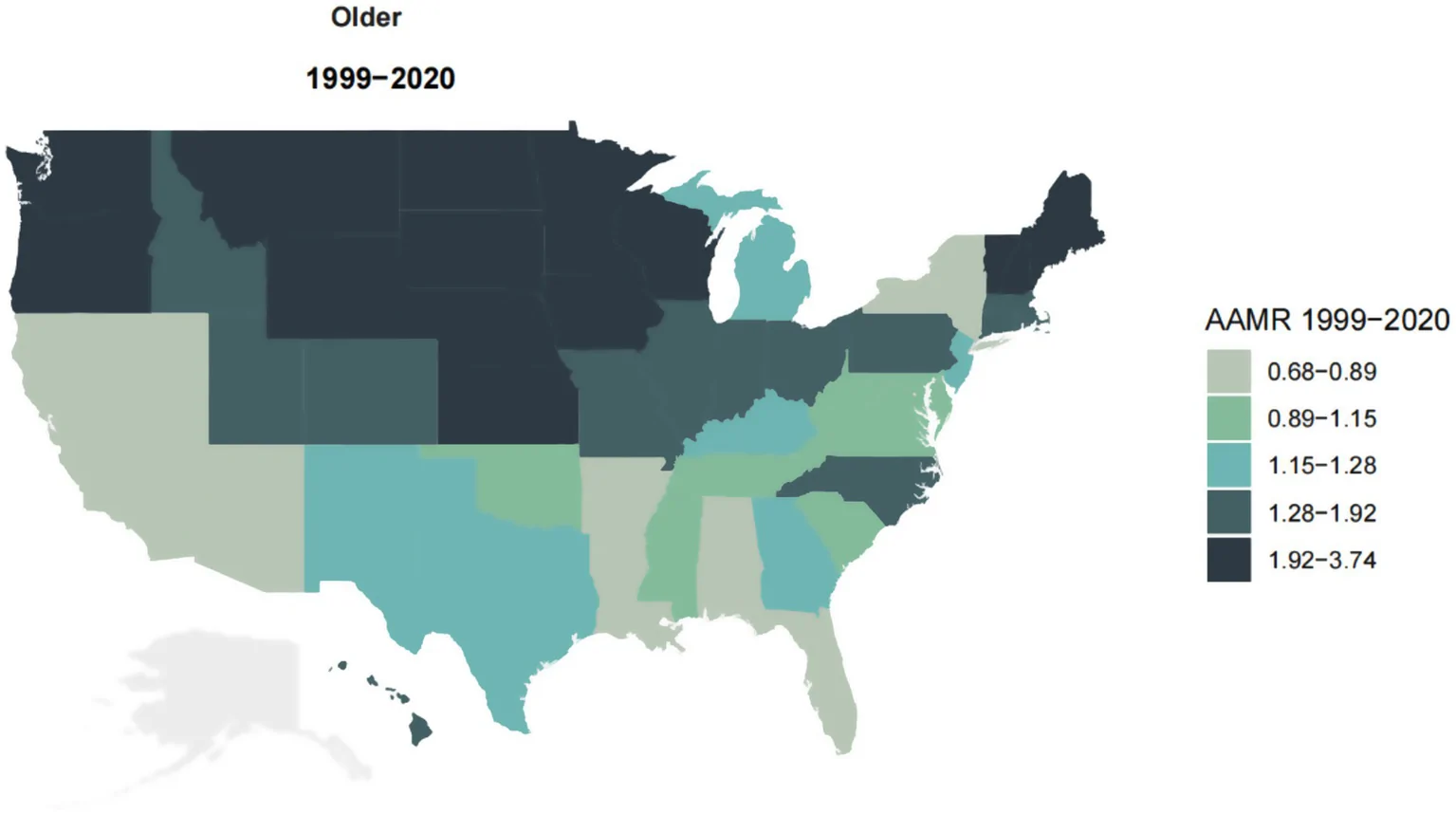
State-level recorded mortality with depression as the underlying cause among U.S. adults aged ≥65 years, 1999–2020.

### Age-specific mortality patterns

Recorded age-specific mortality increased markedly with age in both 1999 and 2020 (Supplementary Figures S1 and S2). In 1999, rates were 0.35, 2.12, and 8.96 per 100,000 population among adults aged 65–74, 75–84, and ≥85 years, respectively; in 2020, the corresponding rates were 0.36, 0.99, and 3.80 (Supplementary Table S2).

The largest relative reduction occurred among adults aged ≥85 years (57.6%), corresponding to an absolute decrease of 5.16 deaths per 100,000, followed by those aged 75–84 years (53.2%; absolute decrease, 1.13 per 100,000). Rates among adults aged 65–74 years remained relatively stable. Women generally had higher recorded rates than men within the same age groups; among adults aged ≥85 years in 2020, rates were 4.22 in women and 3.07 in men. The longitudinal patterns showed reductions in the two oldest age groups and year-to-year fluctuations, particularly among adults aged 65–74 years (Supplementary Figure S2).

Annual recorded death counts and AAMRs generally declined, with fluctuations and an increase in 2020 (Supplementary Table S3 and Figure S3). The ≥85-year group remained the largest age category by recorded deaths, but its share of overall deaths decreased from 372/696 (53.4%) in 1999 to 253/534 (47.4%) in 2020. Thus, the oldest group did not account for more than half of recorded deaths in every year. The share among adults aged 65–74 years increased from 9.3 to 22.1%. Supplementary Figure S3 presents death-count-based age distributions separately from mortality rates.

### Temporal trends in age-adjusted mortality rates

Joinpoint regression identified three inflection points in the overall temporal trend (Figure 2A and Table 2). The AAMR remained relatively stable from 1999 to 2003 (annual percent change [APC], 1.16%; 95% CI, −1.76 to 6.15%; *p* = 0.369), declined sharply from 2003 to 2007 (APC, −11.55%; 95% CI, −15.23% to −8.27%; *p* < 0.001), and continued to decrease from 2007 to 2018 (APC, −3.58%; 95% CI, −5.50% to −2.12%; *p* = 0.012). A nonsignificant increase was observed from 2018 to 2020 (APC, 7.75%; 95% CI, −1.71 to 13.11%; *p* = 0.132). The overall average annual percent change (AAPC) was −3.26% (95% CI, −3.94% to −2.73%; *p* < 0.001).

**Figure 2:**
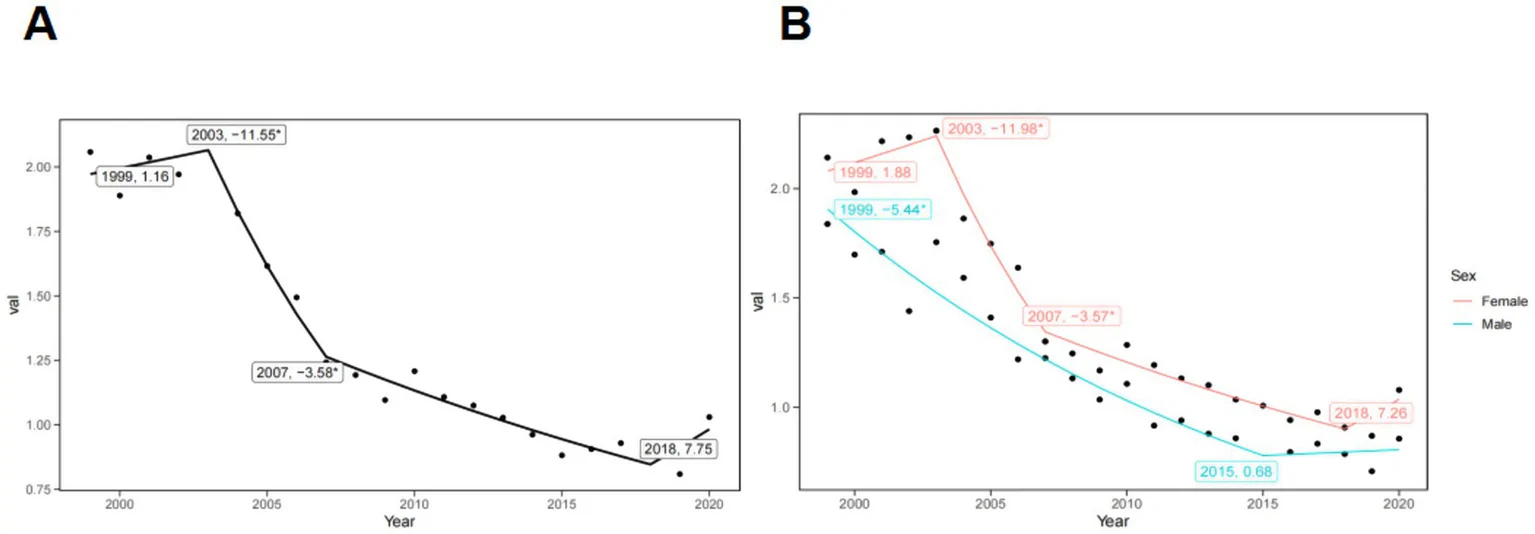
Joinpoint regression of recorded depression-related age-adjusted mortality rates, 1999–2020. **(A)** Overall population; **(B)** Women and men.

**Table 2:** Joinpoint analysis of recorded depression-related AAMR trends by demographic and geographic characteristics, United States, 1999–2020.

Population	Segment	Start	End	APC (%) (95% CI)	*p* value	AAPC (%) (95% CI)	*p* value
Overall	1	1999	2003	1.16 (−1.76 to 6.15)	0.369	−3.26 (−3.94 to −2.73)	<0.001
	2	2003	2007	−11.55 (−15.23 to −8.27)	<0.001		
	3	2007	2018	−3.58 (−5.50 to −2.12)	0.012		
	4	2018	2020	7.75 (−1.71 to 13.11)	0.132		
Sex							
Female	1	1999	2003	1.88 (−1.04 to 6.06)	0.164	−3.26 (−3.90 to −2.77)	<0.001
	2	2003	2007	−11.98 (−15.57 to −8.75)	<0.001		
	3	2007	2018	−3.58 (−5.66 to −2.23)	0.012		
	4	2018	2020	7.26 (−1.96 to 12.67)	0.155		
Male	1	1999	2015	−5.44 (−8.41 to −4.61)	0.016	−4.02 (−4.97 to −3.36)	<0.001
	2	2015	2020	0.68 (−3.94 to 12.01)	0.741		
Urbanization							
Metropolitan	1	1999	2003	1.56 (−0.90 to 5.02)	0.172	−3.46 (−3.85 to −3.01)	<0.001
	2	2003	2007	−12.08 (−15.33 to −9.29)	<0.001		
	3	2007	2015	−4.02 (−6.43 to −2.41)	<0.001		
	4	2015	2020	0.84 (−1.42 to 6.72)	0.414		
Nonmetropolitan	1	1999	2020	−4.22 (−5.29 to −3.27)	<0.001	−4.22 (−5.29 to −3.27)	<0.001
Census region							
Northeast	1	1999	2012	−4.69 (−8.06 to −3.22)	0.001	−2.14 (−3.13 to −1.19)	<0.001
	2	2012	2020	2.14 (−0.97 to 11.45)	0.171		
Midwest	1	1999	2020	−5.54 (−6.32 to −4.88)	<0.001	−5.54 (−6.32 to −4.88)	<0.001
South	1	1999	2003	3.52 (−0.73 to 12.85)	0.108	−4.10 (−4.69 to −3.23)	<0.001
	2	2003	2007	−12.97 (−18.32 to −7.73)	<0.001		
	3	2007	2020	−3.49 (−4.58 to −1.14)	0.026		
West	1	1999	2016	−5.78 (−13.93 to 2.94)	0.054	−4.00 (−5.76 to −2.65)	<0.001
	2	2016	2020	3.95 (−5.27 to 25.54)	0.563		
White (race)	1	1999	2020	−4.71 (−5.48 to −3.99)	<0.001	−4.71 (−5.48 to −3.99)	<0.001

APC and AAPC values are percentages; 95% CIs are shown in parentheses. AAPCs summarize 1999–2020. Segment-specific *p* values test the corresponding APC; AAPC *p* values refer to the full-period trend. All estimates and *p* values are retained from the supplied Joinpoint output; displayed estimates are rounded to two decimal places. Blank cells indicate continuation of the preceding group. These segmented estimates are distinct from the unsegmented endpoint-exclusion sensitivity analysis in Supplementary Table S6. APC, annual percent change; AAPC, average annual percent change; CI, confidence interval.

Sex-specific analyses demonstrated distinct temporal patterns (Figure 2B and Supplementary Table S4). Among women, the AAMR decreased from 2.14 per 100,000 population in 1999 to 1.08 in 2020. The trend was characterized by a marked decline from 2003 to 2007 (APC, −11.98%; *p* < 0.001), a continued reduction from 2007 to 2018 (APC, −3.58%; *p* = 0.012), and a nonsignificant increase after 2018. Among men, the AAMR declined from 1.84 in 1999 to 0.86 in 2020, with a significant reduction from 1999 to 2015 (APC, −5.44%; *p* = 0.016) followed by a plateau from 2015 to 2020 (APC, 0.68%; *p* = 0.741). The AAPC was −3.26% among women and −4.02% among men (both *p* < 0.001).

A significant long-term decline was observed in both metropolitan and nonmetropolitan areas. The AAPC was −3.46% in metropolitan areas and −4.22% in nonmetropolitan areas (both *p* < 0.001). Declining trends were also observed across all U.S. Census regions, with the steepest AAPC in the Midwest (−5.54%), followed by the South (−4.10%), the West (−4.00%), and the Northeast (−2.14%) (all *p* < 0.001).

### Sensitivity analysis excluding 2020

In the descriptive endpoint comparison, overall AAMR decreased from 2.06 per 100,000 in 1999 to 0.81 in 2019, a 60.7% reduction. Rates also declined before 2020 among women (2.14–0.87) and men (1.84–0.71). In 2020, overall AAMR was 1.03, 27.3% above the 2019 value but 50.0% below the 1999 value (Supplementary Table S5). The annual series declined from 0.88 in 2018 to 0.81 in 2019 before increasing in 2020; this was not a sustained annual rise throughout 2018–2020.

The unsegmented log-linear APC was −4.98% (95% CI, −5.71% to −4.26%; *p* < 0.001) for 1999–2019, compared with −4.59% (95% CI, −5.52% to −3.66%; *p* < 0.001) for 1999–2020. Significant downward trends persisted among women and men after excluding 2020 (Supplementary Table S6).

### Sensitivity analysis using multiple-cause-of-death data

The broader definition identified 236,831 deaths with a recorded mention of depression, compared with 11,858 under the underlying-cause definition, a 19.97-fold difference. The primary definition represented 5.01% of death certificates with a documented depression mention. Using the same denominator of 928,476,665 person-years, crude rates were 25.51 and 1.28 per 100,000, respectively (Supplementary Table S7). In the multiple-cause analysis, age-specific rates increased from 8.44 in 1999 to 12.65 in 2020 among adults aged 65–74 years, while rates changed from 31.40 to 30.97 among those aged 75–84 years and from 107.15 to 102.43 among those aged ≥85 years (Supplementary Table S8).

The three-age-band standardized multiple-cause rate declined from 28.70 in 1999 to 22.17 in 2016 and then increased to 30.16 in 2020 (Supplementary Table S9 and Figure S4). The age-specific trajectories are shown in Supplementary Figure S5. These late-period patterns were not fully consistent with the decline in recorded underlying-cause mortality.

### ARIMA-based mortality projections

The retained R-based ARIMA models projected a continued decline in recorded underlying-cause mortality through 2030 (Figure 3 and Supplementary Table S10). The overall predicted AAMR decreased from 0.98 per 100,000 population in 2021 to 0.54 in 2030. Sex-stratified projections showed a decline from 1.03 to 0.57 among women and from 0.71 to 0.26 among men during the same period. The 95% prediction intervals widened progressively over time, indicating greater uncertainty for longer-term estimates.

**Figure 3:**
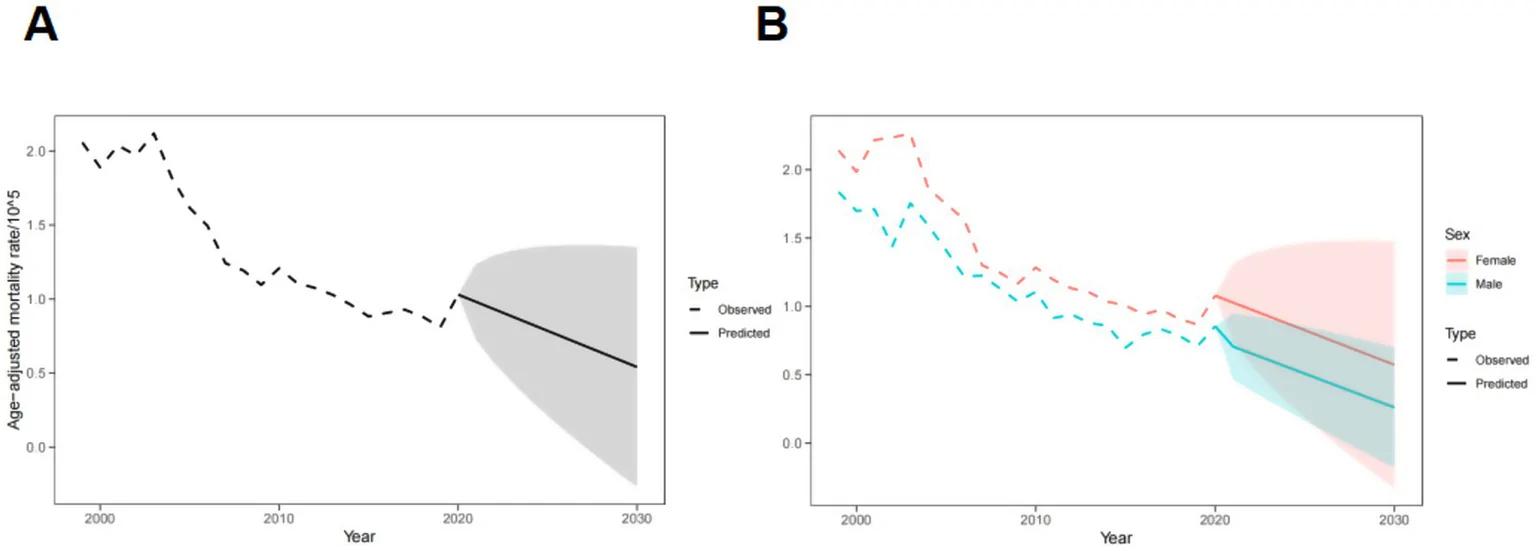
Observed and original R-based ARIMA-projected recorded depression-related AAMRs, 1999–2030. **(A)** Overall population; **(B)** Women and men.

The independent Python refit identified ARIMA(0,1,0) with drift for the overall population and women and ARIMA(0,1,1) with drift for men, with AICc values of −22.258, −17.709, and −22.713, respectively (Supplementary Table S11). Ljung–Box tests at lag 5 were nonsignificant (*p* = 0.839, 0.995, and 0.783), and the residual ACF/PACF plots did not show pronounced remaining serial dependence (Supplementary Figure S6).

In rolling-origin one-year-ahead validation for 2015–2020, ARIMA MAEs were 0.084, 0.077, and 0.140 for the overall, female, and male series, respectively. ARIMA did not consistently outperform damped-trend ETS or the naive benchmark (Supplementary Table S12 and Figure S7).

### Frontier analysis

Frontier analysis demonstrated a nonlinear association between state-level sociodemographic index (SDI) and depression-related AAMR, with considerable heterogeneity among states with similar SDI values (Figure 4). California, Florida, Texas, New York, and Pennsylvania were positioned closest to the low-mortality frontier, whereas Minnesota, Oregon, Iowa, Massachusetts, and Wisconsin exhibited the largest gaps relative to the frontier.

**Figure 4:**
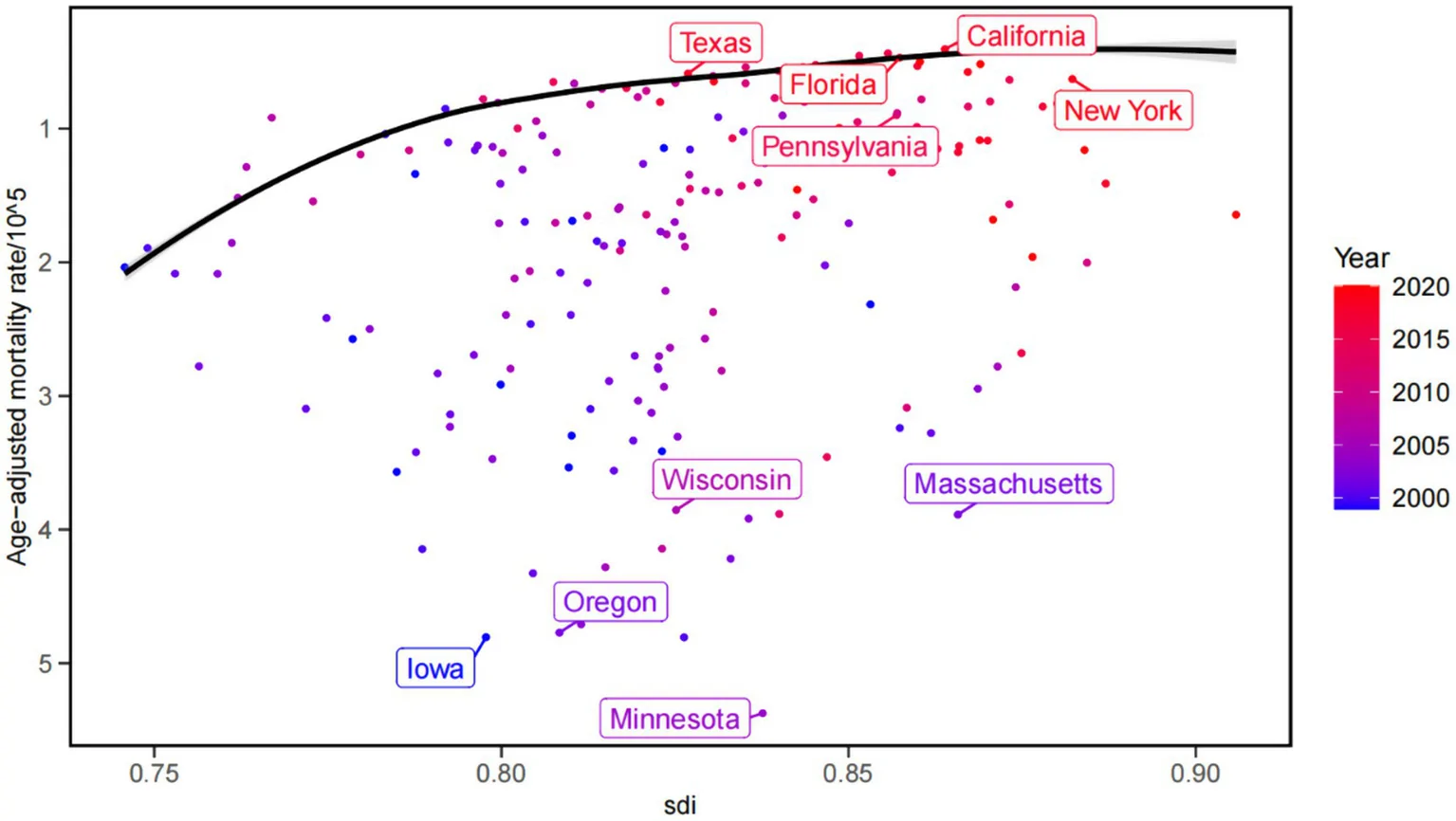
Exploratory Frontier analysis of state-level SDI and recorded depression-related AAMR among U.S. adults aged ≥65 years, 1999–2020.

States with differing reported frontier positions sometimes had similar SDI profiles (Supplementary Table S13). California and Oregon had 2020 SDI values of 0.8690 and 0.8684 and 1999–2020 mean values of 0.8284 and 0.8317. New York and Minnesota likewise had similar 2020 SDI values (0.8840 and 0.8857) and study-period means (0.8555 and 0.8536).

The exploratory state-year smoothing analysis revealed a nonlinear, nonmonotonic relationship between SDI and the recorded depression-related mortality measure (Supplementary Figure S8). The curve increased modestly across the lower SDI range, was relatively elevated at approximately 0.81–0.82, declined toward 0.85–0.87, and showed a slight upward tendency at the highest SDI values. Considerable dispersion occurred among states with similar SDI, and the uncertainty band widened toward both ends of the distribution.

## Discussion

In this national descriptive study of U.S. adults aged ≥65 years during 1999–2020, 11,858 deaths had depression recorded as the underlying cause, corresponding to an overall AAMR of 1.29 per 100,000. Recorded mortality declined most sharply during 2003–2007 and more gradually through 2018; the short upward Joinpoint segment during 2018–2020 was not statistically significant. Higher recorded rates occurred among women, non-Hispanic and White older adults, nonmetropolitan residents, and residents of the Midwest. Age-specific rates remained highest among adults aged ≥85 years despite substantial reductions. The multiple-cause sensitivity analysis identified markedly more deaths and a different late-period pattern. ARIMA projections and Frontier comparisons added exploratory perspectives, but neither established future outcomes, causal mechanisms, policy effectiveness, or preventable mortality.

The sensitivity analysis highlights the dependence of surveillance estimates on the cause-of-death definition. The any-mention definition identified 19.97 times as many death records as the underlying-cause definition and showed an increase in its standardized rate after 2016. The primary decline therefore applies specifically to deaths for which depression was selected as the underlying cause, not to all deaths among individuals with documented depression. Conversely, a depression mention among multiple causes does not establish that depression caused or contributed to every death. Comorbidity, diagnostic recognition, and death certification may contribute to the divergence; both measures may miss undocumented depression.

The overall decline in recorded underlying-cause mortality may be consistent with improvements in the recognition and management of late-life depression over the past two decades. Late-life depression is common, clinically heterogeneous, and often complicated by medical comorbidity, cognitive impairment, functional decline, bereavement, and social isolation (21, 22). Although depression in older adults remains underdiagnosed and undertreated, increasing awareness of geriatric depression, wider use of antidepressant therapy, expansion of psychological interventions, and integration of mental health care into primary care may have contributed to improved outcomes (23). Evidence from a randomized trial demonstrated that collaborative care was more effective than usual care for older adults with depression in primary care settings, supporting the potential value of team-based depression management for this population (24). More recent reviews also suggest that pharmacotherapy, psychotherapy, exercise therapy, and electroconvulsive therapy can be effective depending on depression severity, frailty, medical comorbidity, and patient preference (25).

The sharper decline during 2003–2007 was evident in the annual observations: AAMR decreased from 2.12 to 1.24 per 100,000, approximately 41.4%, and recorded deaths decreased from 762 to 499 (Supplementary Table S3). The IMPACT randomized trial, published in 2002, demonstrated improved depression outcomes with collaborative care and provides relevant clinical context (26). However, trial efficacy does not establish that nationwide implementation of such care caused the mortality decline. The 2003 revision of the U.S. Standard Certificate of Death was implemented progressively across states (27).

The more gradual decline during 2007–2018 may be compatible with persistent underrecognition, multimorbidity, frailty, social isolation, and unequal access to geriatric mental health services (8, 9, 21, 23). Changes in treatment, chronic disease management, diagnostic documentation, and cause-of-death assignment are plausible explanations, but their contributions were not measured.

The clinical importance of depression-related mortality is supported by prior evidence linking late-life depression with adverse survival outcomes. A systematic review and meta-analysis of prospective cohort studies found that late-life depression was associated with increased all-cause and cardiovascular mortality among community-dwelling older adults (4). In addition, an umbrella review reported significant associations between depression and mortality across multiple populations, although it emphasized that causal inference remains limited because much of the evidence is observational and subject to confounding (28). Therefore, the declining recorded underlying-cause rates should not be interpreted as evidence that depression-attributable risk has been reduced to the same extent. Depression may contribute to mortality through multiple pathways, including vascular disease, inflammatory and neuroendocrine dysregulation, sleep disturbance, frailty, poor nutrition, medication nonadherence, reduced physical activity, and delayed care seeking (29).

The nonsignificant upward Joinpoint segment during 2018–2020 requires cautious interpretation. Recorded AAMR declined between 2018 and 2019, with the subsequent increase concentrated in 2020. The pandemic may have affected older adults through disruptions to routine care, depression screening, medication management, psychotherapy, and social support. Isolation, loneliness, bereavement, and difficulties accessing remote care are plausible contributors to vulnerability (30).

Excluding 2020 did not remove the long-term decline, either overall or by sex. Nevertheless, the original Joinpoint segment was short, statistically nonsignificant, and began before the pandemic. Neither that segment nor the 2020 endpoint increase can be attributed specifically to COVID-19. Continued surveillance and analyses incorporating care access, certification practices, and subsequent years are needed to distinguish a transient disturbance from a sustained change.

Women accounted for more than two-thirds of recorded underlying-cause deaths and had a higher recorded AAMR than men. Differences in depressive symptoms, recognition, treatment, documentation, widowhood, disability, and caregiving burden may contribute to this pattern (31). Longer female survival into very old age may affect death counts and population composition, but should not by itself explain a sex difference in age-adjusted rates. Depression among men may also be underrecognized or documented differently.

The racial and ethnic disparities in recorded underlying-cause mortality warrant particular caution. Higher recorded rates among White and non-Hispanic older adults do not necessarily indicate greater depression prevalence or greater depression-attributable mortality risk. Recognition, diagnostic access, cultural expression of distress, stigma, language barriers, healthcare utilization, and death certification may affect whether depression is documented or selected as the underlying cause. Prior studies document disparities in depression diagnosis and treatment among older U.S. adults (32, 33). Lower recorded rates among racial and ethnic minority groups should therefore not be interpreted as evidence of a lower underlying burden. More equitable recognition, culturally responsive care, and improved documentation are important for interpreting surveillance differences.

### Decolonial perspectives and equitable surveillance

A decolonial perspective draws attention to the historical, social, and institutional conditions shaping healthcare access and the production of public health data. The legacies of colonization and dispossession affecting American Indian and Alaska Native communities, together with structural inequities affecting other marginalized populations, provide context for interpreting whose illness becomes visible in clinical and mortality records.

Racial misclassification in American Indian and Alaska Native mortality records has been documented in record-linkage research and can lead to underestimation of recorded deaths (34). Diagnostic and linguistic barriers may further affect depression documentation. These concerns reinforce the need to distinguish recorded mortality from underlying disease burden and to avoid interpreting lower reported rates as evidence of lesser need.

Future surveillance should emphasize culturally appropriate diagnostic practices, community involvement, and data quality. The CARE Principles for Indigenous Data Governance—Collective Benefit, Authority to Control, Responsibility, and Ethics—offer a framework for responsible use of Indigenous data (35).

The pronounced age gradient may reflect a combination of advanced-age vulnerability, accumulated comorbidity, frailty, cognitive and functional impairment, and psychosocial circumstances rather than an isolated effect of chronological aging (9, 21). Cardiovascular disease, diabetes, dementia, dependence on caregivers, bereavement, and institutionalization may interact with depression recognition and management.

High mortality levels and large relative reductions are not contradictory. Rates among adults aged ≥85 years declined by 57.6%, yet remained higher than those in younger groups in 2020. The reductions of 5.16 and 1.13 deaths per 100,000 among the ≥85- and 75–84-year groups were consistent with the overall decline. Their exact contributions to the AAMR change cannot be inferred from these percentages alone, because standard-population weights also matter. Moreover, the ≥85-year group’s share of recorded deaths fell below one-half by 2020, despite remaining the largest age category.

Shared downward trends in metropolitan and nonmetropolitan areas suggest that the recorded decline was not confined to urban populations. Broad developments in depression recognition, primary care integration, and management of chronic disease are possible contributors (8, 23), but changes in documentation may also have affected both settings. Nonmetropolitan residents nevertheless retained a higher overall recorded AAMR (1.72 vs. 1.21 per 100,000). Workforce availability, travel barriers, rural isolation, and population health differences are plausible explanations.

The approximately 5.5-fold variation in reportable state AAMRs and the Frontier analysis indicate substantial geographic heterogeneity. States close to the empirical frontier should not automatically be considered to have better mental health policies or healthcare delivery. SDI summarizes broad development but not psychiatric workforce capacity, treatment quality, antidepressant use, Medicaid-related policies, insurance coverage, long-term care, or comorbidity. California and Oregon, and New York and Minnesota, had similar SDI profiles despite different frontier positions. Age adjustment does not eliminate other differences in case mix or certification.

ARIMA forecasts also require caution. Only 22 annual observations informed a 10-year extrapolation. The 95% PIs are conditional on the fitted forecasting model and do not encompass all model-selection or structural uncertainty, including future changes in coding, policy, treatment, or major disruptions. Negative lower limits are artifacts of unconstrained forecasting and are not plausible mortality outcomes. The independent-refit comparison did not demonstrate consistent superiority over ETS or naive forecasts.

This study has several strengths. It used a national mortality database spanning more than two decades, with age-adjusted and age-specific measures for complementary comparisons. Joinpoint regression characterized changes in temporal trends, and supplementary analyses explicitly evaluated the influence of case definition and the final study year. The incorporation of original forecasts, independent-refit diagnostics, and exploratory Frontier comparisons provided additional descriptive context while making their interpretive limits explicit.

Several limitations should be acknowledged. First, death certificate data are subject to underrecognition of depression, incomplete documentation, and variation in certification. The primary underlying-cause definition captured a much narrower subset than the any-mention definition, while neither identifies the total number of deaths causally attributable to depression.

Second, small subgroup counts and missing or unreliable rates limit inference for smaller populations. Racial and ethnic misclassification and unequal access to diagnosis may affect representation in mortality surveillance. The open-ended ≥85-year group may obscure important within-group heterogeneity.

Third, these aggregate ecological analyses cannot identify individual-level risk factors or independent effects of chronic disease, antidepressant treatment, adherence, mental health workforce capacity, or Medicaid policies. SDI did not provide multivariable control for these factors. Potential 2020 changes in healthcare access and certification cannot be disentangled by the endpoint comparison or the unsegmented exclusion analysis.

Fourth, Frontier analysis and selected-state SDI comparisons were exploratory and did not establish causal mechanisms, superior health-system performance, or preventable mortality. Unmeasured population composition, comorbidity, access to care, treatment, and documentation may influence geographic gaps.

Finally, ARIMA extrapolations were based on short, univariate annual series. Residual diagnostics do not establish external predictive validity, and six 1-year validation forecasts provide limited information. Forecast intervals do not fully incorporate model-selection or future structural uncertainty, and unconstrained lower limits can fall below zero.

## Conclusion

Mortality with depression recorded as the underlying cause among U.S. adults aged ≥65 years declined during 1999–2020, with persistent demographic and geographic differences. The decline remained after excluding 2020, but the broader any-mention definition identified many more deaths and a different late-period pattern. Continued surveillance, improved diagnostic and mortality documentation, culturally responsive care, and meaningful involvement of historically marginalized communities are important priorities. Linked clinical data and longitudinal area-level studies are needed to clarify the determinants of these disparities.

## Data Availability

Publicly available datasets were analyzed in this study. This data can be found at: The survey data are publicly available on the internet for data users and researchers throughout the world (https://wonder.cdc.gov/).
