# Supplementary material for: Trends and disparities in depression-related mortality among older adults in the United States, 1999–2020
Source: Front Public Health. 2026 Oct 28;14:1908154. doi: 10.3389/fpubh.2026.1908154 (PMC13751397; doi:10.3389/fpubh.2026.1908154)
Supplement: Supplementary file 1 [file Data_Sheet_1.docx]

**Supplementary Material**

## Table inventory

S1. State-level recorded mortality with depression as the underlying cause among adults aged ≥65 years, United States, 1999–2020.

S2. Annual age- and sex-specific recorded mortality with depression as the underlying cause, 1999–2020.

S3. Annual recorded deaths and crude and age-adjusted mortality rates, overall and by sex, 1999–2020.

S4. Joinpoint segment estimates for recorded depression-related AAMRs, overall and by sex, 1999–2020.

S5. Descriptive comparison of recorded underlying-cause AAMRs in 1999, 2019, and 2020, overall and by sex.

S6. Sensitivity analysis of long-term recorded underlying-cause mortality trends with and without 2020.

S7. Comparison of underlying-cause and any-mention multiple-cause mortality indicators among U.S. adults aged ≥65 years, 1999–2020.

S8. Age-specific any-mention MCOD mortality rates in 1999 and 2020 among adults aged ≥65 years.

S9. Annual death counts and mortality rates with any mention of depression among U.S. adults aged ≥65 years, 1999–2020.

S10. Original R-based ARIMA-projected recorded depression-related AAMRs, 2021–2030.

S11. Independent Python-refit ARIMA specifications and residual diagnostics.

S12. Exploratory rolling-origin one-year-ahead forecast accuracy, 2015–2020.

S13. SDI profiles of selected states with different reported frontier positions, 1999–2020.

**Supplementary Table S1. State-level recorded mortality with depression as the underlying cause among adults aged ≥65 years, United States, 1999–2020.**

| **State** | **Deaths** | **Person-years** | **Crude rate (95% CI)** | **SE, crude** | **AAMR (95% CI)** | **SE, AAMR** | **Share of all deaths (%)** |
| --- | --- | --- | --- | --- | --- | --- | --- |
| Alabama | 104 | 15,031,638 | 0.69 (0.56–0.82) | 0.068 | 0.77 (0.62–0.92) | 0.076 | 0.88% |
| Alaska | 15 | 1,287,383 | NR (0.65–1.92) | 0.301 | NR (0.78–2.38) | 0.373 | 0.13% |
| Arizona | 170 | 20,479,747 | 0.83 (0.70–0.95) | 0.064 | 0.89 (0.75–1.02) | 0.069 | 1.43% |
| Arkansas | 79 | 9,499,821 | 0.83 (0.66–1.04) | 0.094 | 0.87 (0.69–1.08) | 0.097 | 0.67% |
| California | 726 | 98,463,455 | 0.74 (0.68–0.79) | 0.027 | 0.76 (0.70–0.81) | 0.028 | 6.12% |
| Colorado | 208 | 12,878,798 | 1.61 (1.40–1.83) | 0.112 | 1.71 (1.47–1.94) | 0.119 | 1.75% |
| Connecticut | 169 | 11,551,674 | 1.46 (1.24–1.68) | 0.113 | 1.31 (1.11–1.51) | 0.102 | 1.43% |
| Delaware | 32 | 2,998,633 | 1.07 (0.73–1.51) | 0.189 | 1.15 (0.78–1.62) | 0.204 | 0.27% |
| District of Columbia | 11 | 1,605,482 | NR (0.34–1.23) | 0.207 | NR (0.32–1.16) | 0.196 | 0.09% |
| Florida | 562 | 75,812,682 | 0.74 (0.68–0.80) | 0.031 | 0.74 (0.68–0.81) | 0.031 | 4.74% |
| Georgia | 250 | 23,851,312 | 1.05 (0.92–1.18) | 0.066 | 1.18 (1.04–1.33) | 0.075 | 2.11% |
| Hawaii | 59 | 4,486,904 | 1.31 (1.00–1.70) | 0.171 | 1.28 (0.97–1.66) | 0.169 | 0.50% |
| Idaho | 78 | 4,493,567 | 1.74 (1.37–2.17) | 0.197 | 1.80 (1.42–2.25) | 0.205 | 0.66% |
| Illinois | 620 | 37,010,208 | 1.68 (1.54–1.81) | 0.067 | 1.62 (1.49–1.75) | 0.065 | 5.23% |
| Indiana | 251 | 19,246,805 | 1.30 (1.14–1.47) | 0.082 | 1.29 (1.13–1.45) | 0.082 | 2.12% |
| Iowa | 278 | 10,382,479 | 2.68 (2.36–2.99) | 0.161 | 2.35 (2.07–2.63) | 0.143 | 2.34% |
| Kansas | 208 | 8,664,199 | 2.40 (2.07–2.73) | 0.166 | 2.15 (1.85–2.44) | 0.150 | 1.75% |
| Kentucky | 142 | 13,201,139 | 1.08 (0.90–1.25) | 0.090 | 1.16 (0.97–1.35) | 0.098 | 1.20% |
| Louisiana | 83 | 13,009,017 | 0.64 (0.51–0.79) | 0.070 | 0.68 (0.54–0.84) | 0.074 | 0.70% |
| Maine | 107 | 4,871,522 | 2.20 (1.78–2.61) | 0.212 | 2.19 (1.77–2.61) | 0.212 | 0.90% |
| Maryland | 162 | 16,313,687 | 0.99 (0.84–1.15) | 0.078 | 1.02 (0.86–1.18) | 0.081 | 1.37% |
| Massachusetts | 433 | 20,988,975 | 2.06 (1.87–2.26) | 0.099 | 1.90 (1.72–2.08) | 0.092 | 3.65% |
| Michigan | 382 | 31,211,334 | 1.22 (1.10–1.35) | 0.063 | 1.21 (1.09–1.33) | 0.062 | 3.22% |
| Minnesota | 410 | 15,742,850 | 2.60 (2.35–2.86) | 0.129 | 2.36 (2.13–2.59) | 0.117 | 3.46% |
| Mississippi | 87 | 8,729,223 | 1.00 (0.80–1.23) | 0.107 | 1.06 (0.85–1.31) | 0.114 | 0.73% |
| Missouri | 295 | 19,094,181 | 1.54 (1.37–1.72) | 0.090 | 1.51 (1.34–1.69) | 0.088 | 2.49% |
| Montana | 74 | 3,386,989 | 2.19 (1.72–2.74) | 0.254 | 2.22 (1.74–2.79) | 0.259 | 0.62% |
| Nebraska | 155 | 5,670,397 | 2.73 (2.30–3.16) | 0.220 | 2.45 (2.06–2.84) | 0.199 | 1.31% |
| Nevada | 51 | 7,505,328 | 0.68 (0.51–0.89) | 0.095 | 0.84 (0.62–1.10) | 0.118 | 0.43% |
| New Hampshire | 129 | 4,150,226 | 3.11 (2.57–3.65) | 0.274 | 3.14 (2.60–3.69) | 0.278 | 1.09% |
| New Jersey | 350 | 27,204,021 | 1.29 (1.15–1.42) | 0.069 | 1.26 (1.12–1.39) | 0.068 | 2.95% |
| New Mexico | 72 | 6,206,491 | 1.16 (0.91–1.46) | 0.137 | 1.21 (0.94–1.52) | 0.143 | 0.61% |
| New York | 517 | 60,246,984 | 0.86 (0.78–0.93) | 0.038 | 0.82 (0.75–0.89) | 0.036 | 4.36% |
| North Carolina | 341 | 28,338,738 | 1.20 (1.08–1.33) | 0.065 | 1.32 (1.18–1.46) | 0.071 | 2.88% |
| North Dakota | 79 | 2,235,247 | 3.53 (2.80–4.41) | 0.398 | 2.98 (2.35–3.72) | 0.340 | 0.67% |
| Ohio | 694 | 37,241,492 | 1.86 (1.73–2.00) | 0.071 | 1.81 (1.68–1.95) | 0.069 | 5.85% |
| Oklahoma | 105 | 11,508,359 | 0.91 (0.74–1.09) | 0.089 | 0.93 (0.75–1.11) | 0.091 | 0.89% |
| Oregon | 361 | 12,418,984 | 2.91 (2.61–3.21) | 0.153 | 2.91 (2.61–3.21) | 0.154 | 3.04% |
| Pennsylvania | 640 | 45,068,602 | 1.42 (1.31–1.53) | 0.056 | 1.33 (1.22–1.43) | 0.053 | 5.40% |
| Rhode Island | 67 | 3,529,499 | 1.90 (1.47–2.41) | 0.232 | 1.64 (1.26–2.08) | 0.202 | 0.56% |
| South Carolina | 138 | 14,642,206 | 0.94 (0.79–1.10) | 0.080 | 1.05 (0.88–1.23) | 0.090 | 1.16% |
| South Dakota | 61 | 2,694,651 | 2.26 (1.73–2.91) | 0.290 | 2.02 (1.54–2.61) | 0.262 | 0.51% |
| Tennessee | 199 | 19,431,886 | 1.02 (0.88–1.17) | 0.073 | 1.09 (0.93–1.24) | 0.077 | 1.68% |
| Texas | 646 | 60,198,208 | 1.07 (0.99–1.16) | 0.042 | 1.16 (1.07–1.25) | 0.046 | 5.45% |
| Utah | 72 | 5,738,248 | 1.25 (0.98–1.58) | 0.148 | 1.33 (1.04–1.67) | 0.157 | 0.61% |
| Vermont | 79 | 2,102,797 | 3.76 (2.97–4.68) | 0.423 | 3.74 (2.95–4.66) | 0.422 | 0.67% |
| Virginia | 231 | 22,472,390 | 1.03 (0.90–1.16) | 0.068 | 1.08 (0.94–1.22) | 0.071 | 1.95% |
| Washington | 373 | 19,236,376 | 1.94 (1.74–2.14) | 0.100 | 1.92 (1.73–2.12) | 0.100 | 3.15% |
| West Virginia | 71 | 6,797,832 | 1.04 (0.82–1.32) | 0.124 | 1.11 (0.87–1.41) | 0.132 | 0.60% |
| Wisconsin | 386 | 17,924,268 | 2.15 (1.94–2.37) | 0.110 | 2.02 (1.82–2.22) | 0.103 | 3.26% |
| Wyoming | 46 | 1,619,731 | 2.84 (2.08–3.79) | 0.419 | 3.07 (2.25–4.09) | 0.453 | 0.39% |
| Total | 11,858 | 928,476,665 | 1.28 (1.25–1.30) | 0.012 | 1.29 (1.27–1.32) | 0.012 | 100.00% |

**Supplementary Table S2. Annual age- and sex-specific recorded mortality with depression as the underlying cause, 1999–2020.**

| **Year** | **Age group** | **Sex** | **Deaths** | **Population** | **Age-specific rate (95% CI)** | **SE** | **Share of all deaths (%)** |
| --- | --- | --- | --- | --- | --- | --- | --- |
| 1999 | 65-74 years | Female | 38 | 10,125,424 | 0.38 (0.27–0.52) | 0.061 | 0.32% |
| 1999 | 65-74 years | Male | 27 | 8,293,485 | 0.33 (0.21–0.47) | 0.063 | 0.23% |
| 1999 | 65-74 years | Overall | 65 | 18,418,909 | 0.35 (0.27–0.45) | 0.044 | 0.55% |
| 1999 | 75-84 years | Female | 172 | 7,411,198 | 2.32 (1.97–2.67) | 0.177 | 1.45% |
| 1999 | 75-84 years | Male | 87 | 4,813,716 | 1.81 (1.45–2.23) | 0.194 | 0.73% |
| 1999 | 75-84 years | Overall | 259 | 12,224,914 | 2.12 (1.86–2.38) | 0.132 | 2.18% |
| 1999 | ≥85 years | Female | 270 | 2,960,708 | 9.12 (8.03–10.21) | 0.555 | 2.28% |
| 1999 | ≥85 years | Male | 102 | 1,193,310 | 8.55 (6.89–10.21) | 0.846 | 0.86% |
| 1999 | ≥85 years | Overall | 372 | 4,154,018 | 8.96 (8.04–9.87) | 0.464 | 3.14% |
| 1999 | Overall ≥65 | Overall | 696 | 34,797,841 | 2.00 (1.85–2.15) | 0.076 | 5.87% |
| 2000 | 65-74 years | Female | 31 | 10,087,712 | 0.31 (0.21–0.44) | 0.055 | 0.26% |
| 2000 | 65-74 years | Male | 25 | 8,303,274 | 0.30 (0.20–0.44) | 0.060 | 0.21% |
| 2000 | 65-74 years | Overall | 56 | 18,390,986 | 0.30 (0.23–0.40) | 0.041 | 0.47% |
| 2000 | 75-84 years | Female | 145 | 7,481,827 | 1.94 (1.62–2.25) | 0.161 | 1.22% |
| 2000 | 75-84 years | Male | 63 | 4,879,353 | 1.29 (0.99–1.65) | 0.163 | 0.53% |
| 2000 | 75-84 years | Overall | 208 | 12,361,180 | 1.68 (1.45–1.91) | 0.117 | 1.75% |
| 2000 | ≥85 years | Female | 282 | 3,012,589 | 9.36 (8.27–10.45) | 0.557 | 2.38% |
| 2000 | ≥85 years | Male | 108 | 1,226,998 | 8.80 (7.14–10.46) | 0.847 | 0.91% |
| 2000 | ≥85 years | Overall | 390 | 4,239,587 | 9.20 (8.29–10.11) | 0.466 | 3.29% |
| 2000 | Overall ≥65 | Overall | 654 | 34,991,753 | 1.87 (1.73–2.01) | 0.073 | 5.51% |
| 2001 | 65-74 years | Female | 37 | 10,043,126 | 0.37 (0.26–0.51) | 0.061 | 0.31% |
| 2001 | 65-74 years | Male | 32 | 8,341,053 | 0.38 (0.26–0.54) | 0.068 | 0.27% |
| 2001 | 65-74 years | Overall | 69 | 18,384,179 | 0.38 (0.29–0.47) | 0.045 | 0.58% |
| 2001 | 75-84 years | Female | 165 | 7,589,733 | 2.17 (1.84–2.51) | 0.169 | 1.39% |
| 2001 | 75-84 years | Male | 77 | 5,003,885 | 1.54 (1.21–1.92) | 0.175 | 0.65% |
| 2001 | 75-84 years | Overall | 242 | 12,593,618 | 1.92 (1.68–2.16) | 0.124 | 2.04% |
| 2001 | ≥85 years | Female | 305 | 3,051,430 | 9.99 (8.87–11.12) | 0.572 | 2.57% |
| 2001 | ≥85 years | Male | 100 | 1,261,064 | 7.93 (6.38–9.48) | 0.793 | 0.84% |
| 2001 | ≥85 years | Overall | 405 | 4,312,494 | 9.39 (8.48–10.31) | 0.467 | 3.42% |
| 2001 | Overall ≥65 | Overall | 716 | 35,290,291 | 2.03 (1.88–2.18) | 0.076 | 6.04% |
| 2002 | 65-74 years | Female | 32 | 10,016,658 | 0.32 (0.22–0.45) | 0.056 | 0.27% |
| 2002 | 65-74 years | Male | 28 | 8,371,877 | 0.33 (0.22–0.48) | 0.063 | 0.24% |
| 2002 | 65-74 years | Overall | 60 | 18,388,535 | 0.33 (0.25–0.42) | 0.042 | 0.51% |
| 2002 | 75-84 years | Female | 182 | 7,664,104 | 2.38 (2.03–2.72) | 0.176 | 1.54% |
| 2002 | 75-84 years | Male | 68 | 5,100,760 | 1.33 (1.03–1.69) | 0.162 | 0.57% |
| 2002 | 75-84 years | Overall | 250 | 12,764,864 | 1.96 (1.72–2.20) | 0.124 | 2.11% |
| 2002 | ≥85 years | Female | 309 | 3,077,446 | 10.04 (8.92–11.16) | 0.571 | 2.61% |
| 2002 | ≥85 years | Male | 86 | 1,291,362 | 6.66 (5.33–8.22) | 0.718 | 0.73% |
| 2002 | ≥85 years | Overall | 395 | 4,368,808 | 9.04 (8.15–9.93) | 0.455 | 3.33% |
| 2002 | Overall ≥65 | Overall | 705 | 35,522,207 | 1.99 (1.84–2.13) | 0.075 | 5.95% |
| 2003 | 65-74 years | Female | 26 | 10,048,202 | 0.26 (0.17–0.38) | 0.051 | 0.22% |
| 2003 | 65-74 years | Male | 26 | 8,452,713 | 0.31 (0.20–0.45) | 0.060 | 0.22% |
| 2003 | 65-74 years | Overall | 52 | 18,500,915 | 0.28 (0.21–0.37) | 0.039 | 0.44% |
| 2003 | 75-84 years | Female | 158 | 7,713,157 | 2.05 (1.73–2.37) | 0.163 | 1.33% |
| 2003 | 75-84 years | Male | 83 | 5,183,281 | 1.60 (1.27–1.99) | 0.176 | 0.70% |
| 2003 | 75-84 years | Overall | 241 | 12,896,438 | 1.87 (1.63–2.10) | 0.120 | 2.03% |
| 2003 | ≥85 years | Female | 357 | 3,127,975 | 11.41 (10.23–12.60) | 0.604 | 3.01% |
| 2003 | ≥85 years | Male | 112 | 1,338,201 | 8.37 (6.82–9.92) | 0.791 | 0.95% |
| 2003 | ≥85 years | Overall | 469 | 4,466,176 | 10.50 (9.55–11.45) | 0.485 | 3.96% |
| 2003 | Overall ≥65 | Overall | 762 | 35,863,529 | 2.12 (1.97–2.28) | 0.077 | 6.43% |
| 2004 | 65-74 years | Female | 34 | 10,110,363 | 0.34 (0.23–0.47) | 0.058 | 0.29% |
| 2004 | 65-74 years | Male | 33 | 8,557,170 | 0.39 (0.27–0.54) | 0.067 | 0.28% |
| 2004 | 65-74 years | Overall | 67 | 18,667,533 | 0.36 (0.28–0.46) | 0.044 | 0.56% |
| 2004 | 75-84 years | Female | 141 | 7,738,742 | 1.82 (1.52–2.12) | 0.153 | 1.19% |
| 2004 | 75-84 years | Male | 63 | 5,251,161 | 1.20 (0.92–1.53) | 0.151 | 0.53% |
| 2004 | 75-84 years | Overall | 204 | 12,989,903 | 1.57 (1.35–1.79) | 0.110 | 1.72% |
| 2004 | ≥85 years | Female | 277 | 3,166,584 | 8.75 (7.72–9.78) | 0.526 | 2.34% |
| 2004 | ≥85 years | Male | 108 | 1,379,299 | 7.83 (6.35–9.31) | 0.753 | 0.91% |
| 2004 | ≥85 years | Overall | 385 | 4,545,883 | 8.47 (7.62–9.31) | 0.432 | 3.25% |
| 2004 | Overall ≥65 | Overall | 656 | 36,203,319 | 1.81 (1.67–1.95) | 0.071 | 5.53% |
| 2005 | 65-74 years | Female | 23 | 10,198,569 | 0.23 (0.14–0.34) | 0.047 | 0.19% |
| 2005 | 65-74 years | Male | 16 | 8,683,128 | NR (0.10–0.30) | 0.046 | 0.14% |
| 2005 | 65-74 years | Overall | 39 | 18,881,697 | 0.21 (0.15–0.28) | 0.033 | 0.33% |
| 2005 | 75-84 years | Female | 149 | 7,753,835 | 1.92 (1.61–2.23) | 0.157 | 1.26% |
| 2005 | 75-84 years | Male | 79 | 5,320,967 | 1.49 (1.18–1.85) | 0.167 | 0.67% |
| 2005 | 75-84 years | Overall | 228 | 13,074,802 | 1.74 (1.52–1.97) | 0.115 | 1.92% |
| 2005 | ≥85 years | Female | 256 | 3,249,455 | 7.88 (6.91–8.84) | 0.492 | 2.16% |
| 2005 | ≥85 years | Male | 91 | 1,443,844 | 6.30 (5.07–7.74) | 0.661 | 0.77% |
| 2005 | ≥85 years | Overall | 347 | 4,693,299 | 7.39 (6.62–8.17) | 0.397 | 2.93% |
| 2005 | Overall ≥65 | Overall | 614 | 36,649,798 | 1.68 (1.54–1.81) | 0.068 | 5.18% |
| 2006 | 65-74 years | Female | 25 | 10,350,638 | 0.24 (0.16–0.36) | 0.048 | 0.21% |
| 2006 | 65-74 years | Male | 17 | 8,852,389 | NR (0.11–0.31) | 0.047 | 0.14% |
| 2006 | 65-74 years | Overall | 42 | 19,203,027 | 0.22 (0.16–0.30) | 0.034 | 0.35% |
| 2006 | 75-84 years | Female | 147 | 7,732,685 | 1.90 (1.59–2.21) | 0.157 | 1.24% |
| 2006 | 75-84 years | Male | 59 | 5,362,466 | 1.10 (0.84–1.42) | 0.143 | 0.50% |
| 2006 | 75-84 years | Overall | 206 | 13,095,151 | 1.57 (1.36–1.79) | 0.110 | 1.74% |
| 2006 | ≥85 years | Female | 235 | 3,347,539 | 7.02 (6.12–7.92) | 0.458 | 1.98% |
| 2006 | ≥85 years | Male | 90 | 1,518,390 | 5.93 (4.77–7.29) | 0.625 | 0.76% |
| 2006 | ≥85 years | Overall | 325 | 4,865,929 | 6.68 (5.95–7.41) | 0.370 | 2.74% |
| 2006 | Overall ≥65 | Overall | 573 | 37,164,107 | 1.54 (1.42–1.67) | 0.064 | 4.83% |
| 2007 | 65-74 years | Female | 22 | 10,595,802 | 0.21 (0.13–0.31) | 0.044 | 0.19% |
| 2007 | 65-74 years | Male | 23 | 9,102,925 | 0.25 (0.16–0.38) | 0.053 | 0.19% |
| 2007 | 65-74 years | Overall | 45 | 19,698,727 | 0.23 (0.17–0.31) | 0.034 | 0.38% |
| 2007 | 75-84 years | Female | 110 | 7,694,491 | 1.43 (1.16–1.70) | 0.136 | 0.93% |
| 2007 | 75-84 years | Male | 50 | 5,392,948 | 0.93 (0.69–1.22) | 0.131 | 0.42% |
| 2007 | 75-84 years | Overall | 160 | 13,087,439 | 1.22 (1.03–1.41) | 0.097 | 1.35% |
| 2007 | ≥85 years | Female | 197 | 3,446,309 | 5.72 (4.92–6.51) | 0.407 | 1.66% |
| 2007 | ≥85 years | Male | 97 | 1,593,236 | 6.09 (4.94–7.43) | 0.618 | 0.82% |
| 2007 | ≥85 years | Overall | 294 | 5,039,545 | 5.83 (5.17–6.50) | 0.340 | 2.48% |
| 2007 | Overall ≥65 | Overall | 499 | 37,825,711 | 1.32 (1.20–1.44) | 0.059 | 4.21% |
| 2008 | 65-74 years | Female | 31 | 11,004,244 | 0.28 (0.19–0.40) | 0.051 | 0.26% |
| 2008 | 65-74 years | Male | 19 | 9,501,435 | NR (0.12–0.31) | 0.046 | 0.16% |
| 2008 | 65-74 years | Overall | 50 | 20,505,679 | 0.24 (0.18–0.32) | 0.034 | 0.42% |
| 2008 | 75-84 years | Female | 86 | 7,656,443 | 1.12 (0.90–1.39) | 0.121 | 0.73% |
| 2008 | 75-84 years | Male | 65 | 5,419,659 | 1.20 (0.93–1.53) | 0.149 | 0.55% |
| 2008 | 75-84 years | Overall | 151 | 13,076,102 | 1.16 (0.97–1.34) | 0.094 | 1.27% |
| 2008 | ≥85 years | Female | 201 | 3,533,026 | 5.69 (4.90–6.48) | 0.401 | 1.70% |
| 2008 | ≥85 years | Male | 81 | 1,662,814 | 4.87 (3.87–6.05) | 0.541 | 0.68% |
| 2008 | ≥85 years | Overall | 282 | 5,195,840 | 5.43 (4.79–6.06) | 0.323 | 2.38% |
| 2008 | Overall ≥65 | Overall | 483 | 38,777,621 | 1.25 (1.13–1.36) | 0.057 | 4.07% |
| 2009 | 65-74 years | Female | 24 | 11,375,157 | 0.21 (0.14–0.31) | 0.043 | 0.20% |
| 2009 | 65-74 years | Male | 24 | 9,857,942 | 0.24 (0.16–0.36) | 0.050 | 0.20% |
| 2009 | 65-74 years | Overall | 48 | 21,233,099 | 0.23 (0.17–0.30) | 0.033 | 0.40% |
| 2009 | 75-84 years | Female | 90 | 7,590,640 | 1.19 (0.95–1.46) | 0.125 | 0.76% |
| 2009 | 75-84 years | Male | 58 | 5,432,135 | 1.07 (0.81–1.38) | 0.140 | 0.49% |
| 2009 | 75-84 years | Overall | 148 | 13,022,775 | 1.14 (0.95–1.32) | 0.093 | 1.25% |
| 2009 | ≥85 years | Female | 189 | 3,631,954 | 5.20 (4.46–5.95) | 0.379 | 1.59% |
| 2009 | ≥85 years | Male | 76 | 1,735,347 | 4.38 (3.45–5.48) | 0.502 | 0.64% |
| 2009 | ≥85 years | Overall | 265 | 5,367,301 | 4.94 (4.34–5.53) | 0.303 | 2.23% |
| 2009 | Overall ≥65 | Overall | 461 | 39,623,175 | 1.16 (1.06–1.27) | 0.054 | 3.89% |
| 2010 | 65-74 years | Female | 41 | 11,616,910 | 0.35 (0.25–0.48) | 0.055 | 0.35% |
| 2010 | 65-74 years | Male | 21 | 10,096,519 | 0.21 (0.13–0.32) | 0.045 | 0.18% |
| 2010 | 65-74 years | Overall | 62 | 21,713,429 | 0.29 (0.22–0.37) | 0.036 | 0.52% |
| 2010 | 75-84 years | Female | 89 | 7,584,360 | 1.17 (0.94–1.44) | 0.124 | 0.75% |
| 2010 | 75-84 years | Male | 63 | 5,476,762 | 1.15 (0.88–1.47) | 0.145 | 0.53% |
| 2010 | 75-84 years | Overall | 152 | 13,061,122 | 1.16 (0.98–1.35) | 0.094 | 1.28% |
| 2010 | ≥85 years | Female | 196 | 3,703,754 | 5.29 (4.55–6.03) | 0.378 | 1.65% |
| 2010 | ≥85 years | Male | 84 | 1,789,679 | 4.69 (3.74–5.81) | 0.512 | 0.71% |
| 2010 | ≥85 years | Overall | 280 | 5,493,433 | 5.10 (4.50–5.69) | 0.305 | 2.36% |
| 2010 | Overall ≥65 | Overall | 494 | 40,267,984 | 1.23 (1.12–1.33) | 0.055 | 4.17% |
| 2011 | 65-74 years | Female | 30 | 12,005,425 | 0.25 (0.17–0.36) | 0.046 | 0.25% |
| 2011 | 65-74 years | Male | 24 | 10,476,313 | 0.23 (0.15–0.34) | 0.047 | 0.20% |
| 2011 | 65-74 years | Overall | 54 | 22,481,738 | 0.24 (0.18–0.31) | 0.033 | 0.46% |
| 2011 | 75-84 years | Female | 95 | 7,602,197 | 1.25 (1.01–1.53) | 0.128 | 0.80% |
| 2011 | 75-84 years | Male | 44 | 5,573,033 | 0.79 (0.57–1.06) | 0.119 | 0.37% |
| 2011 | 75-84 years | Overall | 139 | 13,175,230 | 1.05 (0.88–1.23) | 0.089 | 1.17% |
| 2011 | ≥85 years | Female | 206 | 3,843,148 | 5.36 (4.63–6.09) | 0.373 | 1.74% |
| 2011 | ≥85 years | Male | 82 | 1,894,025 | 4.33 (3.44–5.37) | 0.478 | 0.69% |
| 2011 | ≥85 years | Overall | 288 | 5,737,173 | 5.02 (4.44–5.60) | 0.296 | 2.43% |
| 2011 | Overall ≥65 | Overall | 481 | 41,394,141 | 1.16 (1.06–1.27) | 0.053 | 4.06% |
| 2012 | 65-74 years | Female | 31 | 12,782,530 | 0.24 (0.17–0.34) | 0.044 | 0.26% |
| 2012 | 65-74 years | Male | 35 | 11,202,862 | 0.31 (0.22–0.43) | 0.053 | 0.29% |
| 2012 | 65-74 years | Overall | 66 | 23,985,392 | 0.28 (0.21–0.35) | 0.034 | 0.56% |
| 2012 | 75-84 years | Female | 88 | 7,624,484 | 1.15 (0.93–1.42) | 0.123 | 0.74% |
| 2012 | 75-84 years | Male | 60 | 5,648,150 | 1.06 (0.81–1.37) | 0.137 | 0.51% |
| 2012 | 75-84 years | Overall | 148 | 13,272,634 | 1.11 (0.94–1.29) | 0.092 | 1.25% |
| 2012 | ≥85 years | Female | 194 | 3,923,297 | 4.95 (4.25–5.64) | 0.355 | 1.64% |
| 2012 | ≥85 years | Male | 62 | 1,964,033 | 3.16 (2.42–4.05) | 0.401 | 0.52% |
| 2012 | ≥85 years | Overall | 256 | 5,887,330 | 4.35 (3.82–4.88) | 0.272 | 2.16% |
| 2012 | Overall ≥65 | Overall | 470 | 43,145,356 | 1.09 (0.99–1.19) | 0.050 | 3.96% |
| 2013 | 65-74 years | Female | 43 | 13,419,124 | 0.32 (0.23–0.43) | 0.049 | 0.36% |
| 2013 | 65-74 years | Male | 25 | 11,797,642 | 0.21 (0.14–0.31) | 0.042 | 0.21% |
| 2013 | 65-74 years | Overall | 68 | 25,216,766 | 0.27 (0.21–0.34) | 0.033 | 0.57% |
| 2013 | 75-84 years | Female | 76 | 7,686,002 | 0.99 (0.78–1.24) | 0.113 | 0.64% |
| 2013 | 75-84 years | Male | 44 | 5,760,517 | 0.76 (0.56–1.02) | 0.115 | 0.37% |
| 2013 | 75-84 years | Overall | 120 | 13,446,519 | 0.89 (0.73–1.05) | 0.081 | 1.01% |
| 2013 | ≥85 years | Female | 190 | 3,999,007 | 4.75 (4.08–5.43) | 0.345 | 1.60% |
| 2013 | ≥85 years | Male | 81 | 2,041,782 | 3.97 (3.15–4.93) | 0.441 | 0.68% |
| 2013 | ≥85 years | Overall | 271 | 6,040,789 | 4.49 (3.95–5.02) | 0.273 | 2.28% |
| 2013 | Overall ≥65 | Overall | 459 | 44,704,074 | 1.03 (0.93–1.12) | 0.048 | 3.87% |
| 2014 | 65-74 years | Female | 31 | 14,049,245 | 0.22 (0.15–0.31) | 0.040 | 0.26% |
| 2014 | 65-74 years | Male | 31 | 12,349,045 | 0.25 (0.17–0.36) | 0.045 | 0.26% |
| 2014 | 65-74 years | Overall | 62 | 26,398,290 | 0.23 (0.18–0.30) | 0.030 | 0.52% |
| 2014 | 75-84 years | Female | 86 | 7,789,312 | 1.10 (0.88–1.36) | 0.119 | 0.73% |
| 2014 | 75-84 years | Male | 45 | 5,893,378 | 0.76 (0.56–1.02) | 0.114 | 0.38% |
| 2014 | 75-84 years | Overall | 131 | 13,682,690 | 0.96 (0.79–1.12) | 0.084 | 1.10% |
| 2014 | ≥85 years | Female | 179 | 4,053,362 | 4.42 (3.77–5.06) | 0.330 | 1.51% |
| 2014 | ≥85 years | Male | 71 | 2,108,869 | 3.37 (2.63–4.25) | 0.400 | 0.60% |
| 2014 | ≥85 years | Overall | 250 | 6,162,231 | 4.06 (3.55–4.56) | 0.257 | 2.11% |
| 2014 | Overall ≥65 | Overall | 443 | 46,243,211 | 0.96 (0.87–1.05) | 0.046 | 3.74% |
| 2015 | 65-74 years | Female | 54 | 14,658,169 | 0.37 (0.28–0.48) | 0.050 | 0.46% |
| 2015 | 65-74 years | Male | 24 | 12,892,348 | 0.19 (0.12–0.28) | 0.038 | 0.20% |
| 2015 | 65-74 years | Overall | 78 | 27,550,517 | 0.28 (0.22–0.35) | 0.032 | 0.66% |
| 2015 | 75-84 years | Female | 75 | 7,899,603 | 0.95 (0.75–1.19) | 0.110 | 0.63% |
| 2015 | 75-84 years | Male | 39 | 6,023,571 | 0.65 (0.46–0.89) | 0.104 | 0.33% |
| 2015 | 75-84 years | Overall | 114 | 13,923,174 | 0.82 (0.67–0.97) | 0.077 | 0.96% |
| 2015 | ≥85 years | Female | 161 | 4,112,863 | 3.92 (3.31–4.52) | 0.309 | 1.36% |
| 2015 | ≥85 years | Male | 68 | 2,174,298 | 3.13 (2.43–3.96) | 0.379 | 0.57% |
| 2015 | ≥85 years | Overall | 229 | 6,287,161 | 3.64 (3.17–4.11) | 0.241 | 1.93% |
| 2015 | Overall ≥65 | Overall | 421 | 47,760,852 | 0.88 (0.80–0.97) | 0.043 | 3.55% |
| 2016 | 65-74 years | Female | 51 | 15,239,221 | 0.34 (0.25–0.44) | 0.047 | 0.43% |
| 2016 | 65-74 years | Male | 30 | 13,391,109 | 0.22 (0.15–0.32) | 0.041 | 0.25% |
| 2016 | 65-74 years | Overall | 81 | 28,630,330 | 0.28 (0.23–0.35) | 0.031 | 0.68% |
| 2016 | 75-84 years | Female | 81 | 8,056,660 | 1.00 (0.80–1.25) | 0.112 | 0.68% |
| 2016 | 75-84 years | Male | 45 | 6,176,874 | 0.73 (0.53–0.97) | 0.109 | 0.38% |
| 2016 | 75-84 years | Overall | 126 | 14,233,534 | 0.89 (0.73–1.04) | 0.079 | 1.06% |
| 2016 | ≥85 years | Female | 145 | 4,155,488 | 3.49 (2.92–4.06) | 0.290 | 1.22% |
| 2016 | ≥85 years | Male | 80 | 2,224,843 | 3.60 (2.85–4.47) | 0.402 | 0.68% |
| 2016 | ≥85 years | Overall | 225 | 6,380,331 | 3.53 (3.07–3.99) | 0.235 | 1.90% |
| 2016 | Overall ≥65 | Overall | 432 | 49,244,195 | 0.88 (0.80–0.96) | 0.042 | 3.64% |
| 2017 | 65-74 years | Female | 53 | 15,806,306 | 0.34 (0.25–0.44) | 0.046 | 0.45% |
| 2017 | 65-74 years | Male | 35 | 13,877,140 | 0.25 (0.18–0.35) | 0.043 | 0.29% |
| 2017 | 65-74 years | Overall | 88 | 29,683,446 | 0.30 (0.24–0.36) | 0.032 | 0.74% |
| 2017 | 75-84 years | Female | 91 | 8,298,676 | 1.10 (0.88–1.35) | 0.115 | 0.77% |
| 2017 | 75-84 years | Male | 50 | 6,407,875 | 0.78 (0.58–1.03) | 0.110 | 0.42% |
| 2017 | 75-84 years | Overall | 141 | 14,706,551 | 0.96 (0.80–1.12) | 0.081 | 1.19% |
| 2017 | ≥85 years | Female | 145 | 4,189,013 | 3.46 (2.90–4.03) | 0.287 | 1.22% |
| 2017 | ≥85 years | Male | 72 | 2,279,669 | 3.16 (2.47–3.98) | 0.372 | 0.61% |
| 2017 | ≥85 years | Overall | 217 | 6,468,682 | 3.35 (2.91–3.80) | 0.228 | 1.83% |
| 2017 | Overall ≥65 | Overall | 446 | 50,858,679 | 0.88 (0.80–0.96) | 0.042 | 3.76% |
| 2018 | 65-74 years | Female | 50 | 16,246,231 | 0.31 (0.23–0.41) | 0.044 | 0.42% |
| 2018 | 65-74 years | Male | 40 | 14,246,085 | 0.28 (0.20–0.38) | 0.044 | 0.34% |
| 2018 | 65-74 years | Overall | 90 | 30,492,316 | 0.29 (0.24–0.36) | 0.031 | 0.76% |
| 2018 | 75-84 years | Female | 73 | 8,659,334 | 0.84 (0.66–1.06) | 0.099 | 0.62% |
| 2018 | 75-84 years | Male | 49 | 6,735,040 | 0.73 (0.54–0.96) | 0.104 | 0.41% |
| 2018 | 75-84 years | Overall | 122 | 15,394,374 | 0.79 (0.65–0.93) | 0.072 | 1.03% |
| 2018 | ≥85 years | Female | 161 | 4,218,810 | 3.82 (3.23–4.41) | 0.301 | 1.36% |
| 2018 | ≥85 years | Male | 73 | 2,325,693 | 3.14 (2.46–3.95) | 0.367 | 0.62% |
| 2018 | ≥85 years | Overall | 234 | 6,544,503 | 3.58 (3.12–4.03) | 0.234 | 1.97% |
| 2018 | Overall ≥65 | Overall | 446 | 52,431,193 | 0.85 (0.77–0.93) | 0.040 | 3.76% |
| 2019 | 65-74 years | Female | 57 | 16,783,854 | 0.34 (0.26–0.44) | 0.045 | 0.48% |
| 2019 | 65-74 years | Male | 28 | 14,699,579 | 0.19 (0.13–0.28) | 0.036 | 0.24% |
| 2019 | 65-74 years | Overall | 85 | 31,483,433 | 0.27 (0.22–0.33) | 0.029 | 0.72% |
| 2019 | 75-84 years | Female | 78 | 8,971,649 | 0.87 (0.69–1.08) | 0.098 | 0.66% |
| 2019 | 75-84 years | Male | 53 | 6,998,223 | 0.76 (0.57–0.99) | 0.104 | 0.45% |
| 2019 | 75-84 years | Overall | 131 | 15,969,872 | 0.82 (0.68–0.96) | 0.072 | 1.10% |
| 2019 | ≥85 years | Female | 135 | 4,228,470 | 3.19 (2.65–3.73) | 0.275 | 1.14% |
| 2019 | ≥85 years | Male | 62 | 2,376,488 | 2.61 (2.00–3.34) | 0.331 | 0.52% |
| 2019 | ≥85 years | Overall | 197 | 6,604,958 | 2.98 (2.57–3.40) | 0.213 | 1.66% |
| 2019 | Overall ≥65 | Overall | 413 | 54,058,263 | 0.76 (0.69–0.84) | 0.038 | 3.48% |
| 2020 | 65-74 years | Female | 73 | 17,365,858 | 0.42 (0.33–0.53) | 0.049 | 0.62% |
| 2020 | 65-74 years | Male | 45 | 15,183,540 | 0.30 (0.22–0.40) | 0.044 | 0.38% |
| 2020 | 65-74 years | Overall | 118 | 32,549,398 | 0.36 (0.30–0.43) | 0.033 | 1.00% |
| 2020 | 75-84 years | Female | 96 | 9,228,272 | 1.04 (0.84–1.27) | 0.106 | 0.81% |
| 2020 | 75-84 years | Male | 67 | 7,223,275 | 0.93 (0.72–1.18) | 0.113 | 0.56% |
| 2020 | 75-84 years | Overall | 163 | 16,451,547 | 0.99 (0.84–1.14) | 0.078 | 1.38% |
| 2020 | ≥85 years | Female | 179 | 4,243,727 | 4.22 (3.60–4.84) | 0.315 | 1.51% |
| 2020 | ≥85 years | Male | 74 | 2,414,693 | 3.06 (2.41–3.85) | 0.356 | 0.62% |
| 2020 | ≥85 years | Overall | 253 | 6,658,420 | 3.80 (3.33–4.27) | 0.239 | 2.13% |
| 2020 | Overall ≥65 | Overall | 534 | 55,659,365 | 0.96 (0.88–1.04) | 0.042 | 4.50% |

**Supplementary Table S3. Annual recorded deaths and crude and age-adjusted mortality rates, overall and by sex, 1999–2020.**

| **Year** | **Sex** | **Deaths** | **Population / person-years** | **Crude rate (95% CI)** | **SE, crude** | **AAMR (95% CI)** | **SE, AAMR** | **Share of all deaths (%)** |
| --- | --- | --- | --- | --- | --- | --- | --- | --- |
| 1999 | Female | 480 | 20,497,330 | 2.34 (2.13–2.55) | 0.107 | 2.14 (1.95–2.33) | 0.098 | 4.05% |
| 1999 | Male | 216 | 14,300,511 | 1.51 (1.31–1.71) | 0.103 | 1.84 (1.59–2.09) | 0.128 | 1.82% |
| 1999 | Overall | 696 | 34,797,841 | 2.00 (1.85–2.15) | 0.076 | 2.06 (1.91–2.21) | 0.078 | 5.87% |
| 2000 | Female | 458 | 20,582,128 | 2.23 (2.02–2.43) | 0.104 | 1.98 (1.80–2.17) | 0.093 | 3.86% |
| 2000 | Male | 196 | 14,409,625 | 1.36 (1.17–1.55) | 0.097 | 1.70 (1.46–1.94) | 0.123 | 1.65% |
| 2000 | Overall | 654 | 34,991,753 | 1.87 (1.73–2.01) | 0.073 | 1.89 (1.74–2.03) | 0.074 | 5.51% |
| 2001 | Female | 507 | 20,684,289 | 2.45 (2.24–2.66) | 0.109 | 2.22 (2.02–2.41) | 0.099 | 4.28% |
| 2001 | Male | 209 | 14,606,002 | 1.43 (1.24–1.62) | 0.099 | 1.71 (1.48–1.95) | 0.120 | 1.76% |
| 2001 | Overall | 716 | 35,290,291 | 2.03 (1.88–2.18) | 0.076 | 2.04 (1.89–2.19) | 0.076 | 6.04% |
| 2002 | Female | 523 | 20,758,208 | 2.52 (2.30–2.73) | 0.110 | 2.23 (2.04–2.43) | 0.098 | 4.41% |
| 2002 | Male | 182 | 14,763,999 | 1.23 (1.05–1.41) | 0.091 | 1.44 (1.23–1.65) | 0.109 | 1.54% |
| 2002 | Overall | 705 | 35,522,207 | 1.99 (1.84–2.13) | 0.075 | 1.97 (1.82–2.12) | 0.074 | 5.95% |
| 2003 | Female | 541 | 20,889,334 | 2.59 (2.37–2.81) | 0.111 | 2.27 (2.07–2.46) | 0.098 | 4.56% |
| 2003 | Male | 221 | 14,974,195 | 1.48 (1.28–1.67) | 0.099 | 1.75 (1.52–1.99) | 0.120 | 1.86% |
| 2003 | Overall | 762 | 35,863,529 | 2.12 (1.97–2.28) | 0.077 | 2.12 (1.97–2.27) | 0.077 | 6.43% |
| 2004 | Female | 452 | 21,015,689 | 2.15 (1.95–2.35) | 0.101 | 1.86 (1.69–2.04) | 0.088 | 3.81% |
| 2004 | Male | 204 | 15,187,630 | 1.34 (1.16–1.53) | 0.094 | 1.59 (1.37–1.81) | 0.113 | 1.72% |
| 2004 | Overall | 656 | 36,203,319 | 1.81 (1.67–1.95) | 0.071 | 1.82 (1.68–1.96) | 0.071 | 5.53% |
| 2005 | Female | 428 | 21,201,859 | 2.02 (1.83–2.21) | 0.098 | 1.75 (1.58–1.91) | 0.085 | 3.61% |
| 2005 | Male | 186 | 15,447,939 | 1.20 (1.03–1.38) | 0.088 | 1.41 (1.21–1.61) | 0.104 | 1.57% |
| 2005 | Overall | 614 | 36,649,798 | 1.68 (1.54–1.81) | 0.068 | 1.62 (1.49–1.74) | 0.065 | 5.18% |
| 2006 | Female | 407 | 21,430,862 | 1.90 (1.72–2.08) | 0.094 | 1.64 (1.48–1.80) | 0.082 | 3.43% |
| 2006 | Male | 166 | 15,733,245 | 1.05 (0.90–1.22) | 0.082 | 1.22 (1.03–1.41) | 0.095 | 1.40% |
| 2006 | Overall | 573 | 37,164,107 | 1.54 (1.42–1.67) | 0.064 | 1.49 (1.37–1.62) | 0.062 | 4.83% |
| 2007 | Female | 329 | 21,736,602 | 1.51 (1.35–1.68) | 0.083 | 1.30 (1.16–1.44) | 0.072 | 2.77% |
| 2007 | Male | 170 | 16,089,109 | 1.06 (0.90–1.22) | 0.081 | 1.23 (1.04–1.41) | 0.094 | 1.43% |
| 2007 | Overall | 499 | 37,825,711 | 1.32 (1.20–1.44) | 0.059 | 1.24 (1.13–1.35) | 0.056 | 4.21% |
| 2008 | Female | 318 | 22,193,713 | 1.43 (1.27–1.59) | 0.080 | 1.25 (1.11–1.39) | 0.071 | 2.68% |
| 2008 | Male | 165 | 16,583,908 | 0.99 (0.84–1.15) | 0.077 | 1.13 (0.96–1.30) | 0.088 | 1.39% |
| 2008 | Overall | 483 | 38,777,621 | 1.25 (1.13–1.36) | 0.057 | 1.19 (1.09–1.30) | 0.055 | 4.07% |
| 2009 | Female | 303 | 22,597,751 | 1.34 (1.19–1.49) | 0.077 | 1.17 (1.03–1.30) | 0.068 | 2.56% |
| 2009 | Male | 158 | 17,025,424 | 0.93 (0.78–1.07) | 0.074 | 1.03 (0.87–1.20) | 0.083 | 1.33% |
| 2009 | Overall | 461 | 39,623,175 | 1.16 (1.06–1.27) | 0.054 | 1.10 (1.00–1.20) | 0.051 | 3.89% |
| 2010 | Female | 326 | 22,905,024 | 1.42 (1.27–1.58) | 0.079 | 1.28 (1.14–1.43) | 0.073 | 2.75% |
| 2010 | Male | 168 | 17,362,960 | 0.97 (0.82–1.11) | 0.075 | 1.11 (0.94–1.27) | 0.086 | 1.42% |
| 2010 | Overall | 494 | 40,267,984 | 1.23 (1.12–1.33) | 0.055 | 1.21 (1.10–1.31) | 0.055 | 4.17% |
| 2011 | Female | 331 | 23,450,770 | 1.41 (1.26–1.56) | 0.078 | 1.19 (1.06–1.32) | 0.066 | 2.79% |
| 2011 | Male | 150 | 17,943,371 | 0.84 (0.70–0.97) | 0.068 | 0.92 (0.77–1.06) | 0.075 | 1.26% |
| 2011 | Overall | 481 | 41,394,141 | 1.16 (1.06–1.27) | 0.053 | 1.11 (1.01–1.21) | 0.051 | 4.06% |
| 2012 | Female | 313 | 24,330,311 | 1.29 (1.14–1.43) | 0.073 | 1.13 (1.00–1.26) | 0.065 | 2.64% |
| 2012 | Male | 157 | 18,815,045 | 0.83 (0.70–0.96) | 0.067 | 0.94 (0.79–1.09) | 0.076 | 1.32% |
| 2012 | Overall | 470 | 43,145,356 | 1.09 (0.99–1.19) | 0.050 | 1.07 (0.98–1.17) | 0.050 | 3.96% |
| 2013 | Female | 309 | 25,104,133 | 1.23 (1.09–1.37) | 0.070 | 1.10 (0.98–1.23) | 0.064 | 2.61% |
| 2013 | Male | 150 | 19,599,941 | 0.77 (0.64–0.89) | 0.062 | 0.88 (0.74–1.02) | 0.072 | 1.26% |
| 2013 | Overall | 459 | 44,704,074 | 1.03 (0.93–1.12) | 0.048 | 1.03 (0.93–1.12) | 0.048 | 3.87% |
| 2014 | Female | 296 | 25,891,919 | 1.14 (1.01–1.27) | 0.066 | 1.03 (0.92–1.16) | 0.061 | 2.50% |
| 2014 | Male | 147 | 20,351,292 | 0.72 (0.61–0.84) | 0.060 | 0.86 (0.72–1.00) | 0.071 | 1.24% |
| 2014 | Overall | 443 | 46,243,211 | 0.96 (0.87–1.05) | 0.046 | 0.96 (0.87–1.05) | 0.046 | 3.74% |
| 2015 | Female | 290 | 26,670,635 | 1.09 (0.96–1.21) | 0.064 | 1.01 (0.89–1.12) | 0.060 | 2.45% |
| 2015 | Male | 131 | 21,090,217 | 0.62 (0.52–0.73) | 0.054 | 0.70 (0.58–0.82) | 0.061 | 1.10% |
| 2015 | Overall | 421 | 47,760,852 | 0.88 (0.80–0.97) | 0.043 | 0.88 (0.80–0.97) | 0.043 | 3.55% |
| 2016 | Female | 277 | 27,451,369 | 1.01 (0.89–1.13) | 0.061 | 0.94 (0.83–1.05) | 0.058 | 2.34% |
| 2016 | Male | 155 | 21,792,826 | 0.71 (0.60–0.82) | 0.057 | 0.80 (0.67–0.92) | 0.065 | 1.31% |
| 2016 | Overall | 432 | 49,244,195 | 0.88 (0.80–0.96) | 0.042 | 0.91 (0.82–0.99) | 0.044 | 3.64% |
| 2017 | Female | 289 | 28,293,995 | 1.02 (0.90–1.14) | 0.060 | 0.98 (0.86–1.09) | 0.058 | 2.44% |
| 2017 | Male | 157 | 22,564,684 | 0.70 (0.59–0.81) | 0.056 | 0.83 (0.70–0.96) | 0.067 | 1.32% |
| 2017 | Overall | 446 | 50,858,679 | 0.88 (0.80–0.96) | 0.042 | 0.93 (0.84–1.02) | 0.044 | 3.76% |
| 2018 | Female | 284 | 29,124,375 | 0.97 (0.86–1.09) | 0.058 | 0.91 (0.80–1.01) | 0.054 | 2.40% |
| 2018 | Male | 162 | 23,306,818 | 0.69 (0.59–0.80) | 0.055 | 0.79 (0.66–0.91) | 0.062 | 1.37% |
| 2018 | Overall | 446 | 52,431,193 | 0.85 (0.77–0.93) | 0.040 | 0.88 (0.80–0.96) | 0.042 | 3.76% |
| 2019 | Female | 270 | 29,983,973 | 0.90 (0.79–1.01) | 0.055 | 0.87 (0.76–0.97) | 0.054 | 2.28% |
| 2019 | Male | 143 | 24,074,290 | 0.59 (0.50–0.69) | 0.050 | 0.71 (0.59–0.82) | 0.060 | 1.21% |
| 2019 | Overall | 413 | 54,058,263 | 0.76 (0.69–0.84) | 0.038 | 0.81 (0.73–0.89) | 0.040 | 3.48% |
| 2020 | Female | 348 | 30,837,857 | 1.13 (1.01–1.25) | 0.060 | 1.08 (0.96–1.19) | 0.058 | 2.94% |
| 2020 | Male | 186 | 24,821,508 | 0.75 (0.64–0.86) | 0.055 | 0.86 (0.73–0.98) | 0.063 | 1.57% |
| 2020 | Overall | 534 | 55,659,365 | 0.96 (0.88–1.04) | 0.042 | 1.03 (0.94–1.12) | 0.045 | 4.50% |
| 1999–2020 | Overall | 11,858 | 928,476,665 | 1.28 (1.25–1.30) | 0.012 | 1.29 (1.27–1.32) | 0.012 | 100.00% |

**Supplementary Table S4. Joinpoint segment estimates for recorded depression-related AAMRs, overall and by sex, 1999–2020.**

| **Population** | **Joinpoints** | **Segment** | **Period** | **APC (%) (95% CI)** | **P value** | **Significant** |
| --- | --- | --- | --- | --- | --- | --- |
| Overall | 3 | 1 | 1999–2003 | 1.16 (−1.76–6.15) | 0.369 | No |
| Overall | 3 | 2 | 2003–2007 | −11.55 (−15.23–−8.27) | <0.001 | Yes |
| Overall | 3 | 3 | 2007–2018 | −3.58 (−5.50–−2.12) | 0.012 | Yes |
| Overall | 3 | 4 | 2018–2020 | 7.75 (−1.71–13.11) | 0.132 | No |
| Female | 3 | 1 | 1999–2003 | 1.88 (−1.04–6.06) | 0.164 | No |
| Female | 3 | 2 | 2003–2007 | −11.98 (−15.57–−8.75) | <0.001 | Yes |
| Female | 3 | 3 | 2007–2018 | −3.58 (−5.66–−2.23) | 0.012 | Yes |
| Female | 3 | 4 | 2018–2020 | 7.26 (−1.96–12.67) | 0.155 | No |
| Male | 1 | 1 | 1999–2015 | −5.44 (−8.41–−4.61) | 0.016 | Yes |
| Male | 1 | 2 | 2015–2020 | 0.68 (−3.94–12.01) | 0.741 | No |

**Supplementary Table S5. Descriptive comparison of recorded underlying-cause AAMRs in 1999, 2019, and 2020, overall and by sex.**

| **Population** | **1999 AAMR (95% CI)** | **2019 AAMR (95% CI)** | **2020 AAMR (95% CI)** | **1999–2019 change** | **2019–2020 change** |
| --- | --- | --- | --- | --- | --- |
| Overall | 2.06 (1.91–2.21) | 0.81 (0.73–0.89) | 1.03 (0.94–1.12) | −60.7% | +27.3% |
| Women | 2.14 (1.95–2.33) | 0.87 (0.76–0.97) | 1.08 (0.96–1.19) | −59.4% | +24.2% |
| Men | 1.84 (1.59–2.09) | 0.71 (0.59–0.82) | 0.86 (0.73–0.98) | −61.5% | +21.1% |

**Supplementary Table S6. Sensitivity analysis of long-term recorded underlying-cause mortality trends with and without 2020.**

| **Group** | **1999–2020 APC (95% CI)** | **1999–2019 APC (95% CI)** | **P value for both** |
| --- | --- | --- | --- |
| Overall | -4.59% (-5.52% to -3.66%) | -4.98% (-5.71% to -4.26%) | <0.001 |
| Female | -4.64% (-5.57% to -3.70%) | -5.01% (-5.75% to -4.26%) | <0.001 |
| Male | -4.53% (-5.28% to -3.78%) | -4.85% (-5.46% to -4.24%) | <0.001 |

**Supplementary Table S7. Comparison of underlying-cause and any-mention multiple-cause mortality indicators among U.S. adults aged ≥65 years, 1999–2020.**

| **Indicator** | **UCOD: F32/F33** | **MCOD: any mention** |
| --- | --- | --- |
| Study population | Age ≥65 years | Age ≥65 years |
| Underlying-cause filter | F32 or F33 | All causes |
| Multiple-cause filter | Not applied | F32 or F33 |
| Deaths | 11,858 | 236,831 |
| Population denominator (person-years) | 928,476,665 | 928,476,665 |
| Crude rate per 100,000 (95% CI) | 1.28 (1.25–1.30) | 25.51 (25.40–25.61) |
| Death count relative to UCOD | 1.00 | 19.97 |

**Supplementary Table S8. Age-specific any-mention MCOD mortality rates in 1999 and 2020 among adults aged ≥65 years.**

| **Age group** | **Deaths, 1999** | **Rate, 1999** | **Deaths, 2020** | **Rate, 2020** | **Change in rate** |
| --- | --- | --- | --- | --- | --- |
| 65-74 years | 1,554 | 8.44 | 4,117 | 12.65 | +49.9% |
| 75-84 years | 3,839 | 31.40 | 5,095 | 30.97 | -1.4% |
| 85+ years | 4,451 | 107.15 | 6,820 | 102.43 | -4.4% |
| Overall (≥65) | 9,844 | 28.29 | 16,032 | 28.80 | +1.8% |

**Supplementary Table S9. Annual death counts and mortality rates with any mention of depression among U.S. adults aged ≥65 years, 1999–2020.**

| **Year** | **Deaths** | **Person-years** | **Crude rate** | **Three-band standardized rate (95% CI)** |
| --- | --- | --- | --- | --- |
| 1999 | 9,844 | 34,797,841 | 28.29 | 28.70 (28.13–29.26) |
| 2000 | 10,481 | 34,991,753 | 29.95 | 30.15 (29.57–30.73) |
| 2001 | 10,692 | 35,290,291 | 30.30 | 30.30 (29.73–30.88) |
| 2002 | 10,899 | 35,522,207 | 30.68 | 30.55 (29.97–31.12) |
| 2003 | 10,727 | 35,863,529 | 29.91 | 29.61 (29.05–30.17) |
| 2004 | 10,146 | 36,203,319 | 28.03 | 27.67 (27.13–28.21) |
| 2005 | 10,394 | 36,649,798 | 28.36 | 27.80 (27.27–28.33) |
| 2006 | 9,994 | 37,164,107 | 26.89 | 26.19 (25.67–26.70) |
| 2007 | 10,164 | 37,825,711 | 26.87 | 26.08 (25.57–26.58) |
| 2008 | 9,999 | 38,777,621 | 25.79 | 25.13 (24.63–25.62) |
| 2009 | 9,389 | 39,623,175 | 23.70 | 23.16 (22.69–23.63) |
| 2010 | 9,588 | 40,267,984 | 23.81 | 23.24 (22.77–23.70) |
| 2011 | 9,901 | 41,394,141 | 23.92 | 23.26 (22.80–23.72) |
| 2012 | 9,951 | 43,145,356 | 23.06 | 22.76 (22.31–23.21) |
| 2013 | 10,211 | 44,704,074 | 22.84 | 22.76 (22.31–23.20) |
| 2014 | 10,356 | 46,243,211 | 22.39 | 22.47 (22.03–22.91) |
| 2015 | 10,619 | 47,760,852 | 22.23 | 22.48 (22.05–22.91) |
| 2016 | 10,715 | 49,244,195 | 21.76 | 22.17 (21.75–22.60) |
| 2017 | 11,737 | 50,858,679 | 23.08 | 23.70 (23.27–24.13) |
| 2018 | 12,160 | 52,431,193 | 23.19 | 23.91 (23.48–24.34) |
| 2019 | 12,832 | 54,058,263 | 23.74 | 24.65 (24.22–25.08) |
| 2020 | 16,032 | 55,659,365 | 28.80 | 30.16 (29.69–30.63) |

**Supplementary Table S10. Original R-based ARIMA-projected recorded depression-related AAMRs, 2021–2030.**

| **Population** | **Year** | **Projected AAMR** | **95% prediction interval** |
| --- | --- | --- | --- |
| Overall | 2021 | 0.98 | 0.72 to 1.24 |
| Overall | 2022 | 0.93 | 0.57 to 1.29 |
| Overall | 2023 | 0.88 | 0.44 to 1.33 |
| Overall | 2024 | 0.83 | 0.32 to 1.35 |
| Overall | 2025 | 0.79 | 0.21 to 1.36 |
| Overall | 2026 | 0.74 | 0.11 to 1.36 |
| Overall | 2027 | 0.69 | 0.01 to 1.36 |
| Overall | 2028 | 0.64 | −0.09 to 1.36 |
| Overall | 2029 | 0.59 | −0.18 to 1.36 |
| Overall | 2030 | 0.54 | −0.27 to 1.35 |
| Female | 2021 | 1.03 | 0.74 to 1.31 |
| Female | 2022 | 0.98 | 0.57 to 1.38 |
| Female | 2023 | 0.93 | 0.43 to 1.42 |
| Female | 2024 | 0.88 | 0.31 to 1.45 |
| Female | 2025 | 0.83 | 0.19 to 1.46 |
| Female | 2026 | 0.78 | 0.08 to 1.48 |
| Female | 2027 | 0.72 | −0.03 to 1.48 |
| Female | 2028 | 0.67 | −0.13 to 1.48 |
| Female | 2029 | 0.62 | −0.23 to 1.48 |
| Female | 2030 | 0.57 | −0.33 to 1.48 |
| Male | 2021 | 0.71 | 0.47 to 0.95 |
| Male | 2022 | 0.66 | 0.39 to 0.93 |
| Male | 2023 | 0.61 | 0.31 to 0.91 |
| Male | 2024 | 0.56 | 0.24 to 0.88 |
| Male | 2025 | 0.51 | 0.16 to 0.85 |
| Male | 2026 | 0.46 | 0.09 to 0.82 |
| Male | 2027 | 0.41 | 0.02 to 0.79 |
| Male | 2028 | 0.36 | −0.04 to 0.76 |
| Male | 2029 | 0.31 | −0.11 to 0.73 |
| Male | 2030 | 0.26 | −0.18 to 0.70 |

**Supplementary Table S11. Independent Python-refit ARIMA specifications and residual diagnostics.**

| **Population** | **Model** | **Drift** | **AICc** | **Ljung–Box Q(5)** | **P value** |
| --- | --- | --- | --- | --- | --- |
| Total | ARIMA(0,1,0) | Yes | -22.258 | 2.076 | 0.839 |
| Female | ARIMA(0,1,0) | Yes | -17.709 | 0.409 | 0.995 |
| Male | ARIMA(0,1,1) | Yes | -22.713 | 1.740 | 0.783 |

**Supplementary Table S12. Exploratory rolling-origin one-year-ahead forecast accuracy, 2015–2020.**

| **Population** | **ARIMA MAE** | **ARIMA RMSE** | **ETS MAE** | **ETS RMSE** | **Naive MAE** | **Naive RMSE** |
| --- | --- | --- | --- | --- | --- | --- |
| Total | 0.084 | 0.128 | 0.081 | 0.120 | 0.078 | 0.103 |
| Female | 0.077 | 0.122 | 0.073 | 0.116 | 0.075 | 0.097 |
| Male | 0.140 | 0.156 | 0.113 | 0.128 | 0.095 | 0.106 |

**Supplementary Table S13. SDI profiles of selected states with different reported frontier positions, 1999–2020.**

| **State** | **SDI 1999** | **SDI 2020** | **Mean SDI 1999–2020** | **Change** |
| --- | --- | --- | --- | --- |
| **States closer to the estimated low-mortality frontier** |  |  |  |  |
| California | 0.7876 | 0.8690 | 0.8284 | +0.0814 |
| Florida | 0.7833 | 0.8603 | 0.8216 | +0.0770 |
| Texas | 0.7458 | 0.8329 | 0.7863 | +0.0871 |
| New York | 0.8234 | 0.8840 | 0.8555 | +0.0606 |
| Pennsylvania | 0.8102 | 0.8708 | 0.8403 | +0.0606 |
| **States with larger estimated frontier gaps** |  |  |  |  |
| Minnesota | 0.8231 | 0.8857 | 0.8536 | +0.0626 |
| Oregon | 0.7956 | 0.8684 | 0.8317 | +0.0728 |
| Iowa | 0.7978 | 0.8627 | 0.8280 | +0.0650 |
| Massachusetts | 0.8532 | 0.9058 | 0.8825 | +0.0527 |
| Wisconsin | 0.8097 | 0.8702 | 0.8388 | +0.0605 |

**Supplementary Figure S1. Sex-specific recorded underlying-cause mortality rates by age group in 1999 and 2020.**

**Supplementary Figure S2. Age- and sex-specific recorded underlying-cause mortality patterns, 1999–2020.**

Legend. (A) Overall age-specific rates in 1999 and 2020; (B) corresponding sex-specific rates; (C) annual trajectories by age group with endpoint rates; (D) annual trajectories by sex and age group with endpoint rates. Rates are per 100,000 population. Shaded bands in the endpoint panels show the displayed uncertainty intervals.

**Supplementary Figure S3. Annual recorded deaths, AAMRs, and death-count-based age distributions, 1999–2020.**

Legend. (A–C) Annual death counts (bars, left axis) and AAMRs (lines, right axis), overall, in women, and in men, respectively. (D–F) Age distributions of recorded deaths overall, in women, and in men.

**Supplementary Figure S4. Annual three-age-band standardized mortality rates with any mention of depression, 1999–2020.**

**Supplementary Figure S5. Annual age-specific mortality rates with any mention of depression, 1999–2020.**

**Supplementary Figure S6. Residual ACF and PACF diagnostics for the independent Python ARIMA refit.**

**Supplementary Figure S7. Exploratory rolling-origin one-year-ahead predictions, 2015–2020.**

**Supplementary Figure S8. Exploratory relationship between SDI and the recorded mortality measure across state-year observations.**
